# MLST and Whole-Genome-Based Population Analysis of *Cryptococcus gattii* VGIII Links Clinical, Veterinary and Environmental Strains, and Reveals Divergent Serotype Specific Sub-populations and Distant Ancestors

**DOI:** 10.1371/journal.pntd.0004861

**Published:** 2016-08-05

**Authors:** Carolina Firacative, Chandler C. Roe, Richard Malik, Kennio Ferreira-Paim, Patricia Escandón, Jane E. Sykes, Laura Rocío Castañón-Olivares, Cudberto Contreras-Peres, Blanca Samayoa, Tania C. Sorrell, Elizabeth Castañeda, Shawn R. Lockhart, David M. Engelthaler, Wieland Meyer

**Affiliations:** 1 Molecular Mycology Research Laboratory, Centre for Infectious Diseases and Microbiology, Sydney Medical School - Westmead Hospital, Marie Bashir Institute for Infectious Diseases and Biosecurity, The University of Sydney, Westmead Millennium Institute, Sydney, Australia; 2 Grupo de Microbiología, Instituto Nacional de Salud, Bogotá, Colombia; 3 Translational Genomics Research Institute, Flagstaff, Arizona, United States of America; 4 Centre for Veterinary Education, The University of Sydney, Sydney, Australia; 5 Infectious Disease Department, Triangulo Mineiro Federal University, Uberaba, Minas Gerais, Brazil; 6 Department of Medicine and Epidemiology, University of California, Davis, Davis, California, United States of America; 7 Departamento de Microbiología y Parasitología, Facultad de Medicina, Universidad National Autónoma de México, Mexico City, Mexico; 8 Instituto Nacional de Diagnóstico y Referencia Epidemiológicos, Mexico City, Mexico; 9 Hospital San Juan de Dios, Guatemala City, Guatemala; 10 Mycotic Diseases Branch, Centers for Disease Control and Prevention, Atlanta, Georgia, United States of America; University of California San Diego School of Medicine, UNITED STATES

## Abstract

The emerging pathogen *Cryptococcus gattii* causes life-threatening disease in immunocompetent and immunocompromised hosts. Of the four major molecular types (VGI-VGIV), the molecular type VGIII has recently emerged as cause of disease in otherwise healthy individuals, prompting a need to investigate its population genetic structure to understand if there are potential genotype-dependent characteristics in its epidemiology, environmental niche(s), host range and clinical features of disease. Multilocus sequence typing (MLST) of 122 clinical, environmental and veterinary *C*. *gattii* VGIII isolates from Australia, Colombia, Guatemala, Mexico, New Zealand, Paraguay, USA and Venezuela, and whole genome sequencing (WGS) of 60 isolates representing all established MLST types identified four divergent sub-populations. The majority of the isolates belong to two main clades, corresponding either to serotype B or C, indicating an ongoing species evolution. Both major clades included clinical, environmental and veterinary isolates. The *C*. *gattii* VGIII population was genetically highly diverse, with minor differences between countries, isolation source, serotype and mating type. Little to no recombination was found between the two major groups, serotype B and C, at the whole and mitochondrial genome level. *C*. *gattii* VGIII is widespread in the Americas, with sporadic cases occurring elsewhere, WGS revealed Mexico and USA as a likely origin of the serotype B VGIII population and Colombia as a possible origin of the serotype C VGIII population. Serotype B isolates are more virulent than serotype C isolates in a murine model of infection, causing predominantly pulmonary cryptococcosis. No specific link between genotype and virulence was observed. Antifungal susceptibility testing against six antifungal drugs revealed that serotype B isolates are more susceptible to azoles than serotype C isolates, highlighting the importance of strain typing to guide effective treatment to improve the disease outcome.

## Author Summary

*Cryptococcus gattii*, which is classically divided into four major molecular types (VGI-VGIV), and two serotypes B and C, is the second most important cause of cryptococcosis. The rising incidence of human and animal cryptococcosis cases caused by molecular type VGIII highlights the need for increased vigilance. In this study, we characterized a large set of *C*. *gattii* VGIII isolates. Genetic analysis revealed four diverging sub-populations, which were primarily associated with serotype B or C, and very likely originated from endemic regions in Colombia, Mexico and the USA. Differences in virulence and antifungal susceptibility between serotypes may result in different disease outcomes since serotype B isolates were more virulent in mice than serotype C isolates, but serotype C isolates were less susceptible to azoles, the primary treatment for uncomplicated cryptococcosis. Identification of cryptococcal serotype and molecular type in clinical practice has the potential to guide treatment regimens and hence reduce morbidity and mortality in both sporadic cases and those associated with outbreaks. Our study significantly contributes to the understanding of the epidemiology, genetics and pathogenesis of *Cryptococcus* and cryptococcosis.

## Introduction

The encapsulated basidiomycetous yeast *Cryptococcus gattii* is the second most important etiological agent of cryptococcosis, next to its sibling species *C*. *neoformans*. Both species can cause central nervous system and pulmonary manifestations [1]. However, *C*. *gattii* has a more limited geographical distribution and is recovered less frequently [2,3]. Initially considered prevalent only in tropical and subtropical regions, *C*. *gattii* emerged in a temperate climatic zone on Vancouver Island, British Columbia, Canada in 1999 and has since extended to the Pacific Northwest and other locations within the USA [4,5]. Despite the fact that the global burden of *C*. *gattii* may still be unrecognized [3], the increasing isolation of uncommon molecular types and extension of their geographic spread defines *C*. *gattii* as an emerging fungal pathogen.

*C*. *gattii* is comprised of two serotypes, B and C. Among them, four major molecular types, (VGI/AFLP4, VGII/AFLP6, VGIII/AFLP5 and VGIV/AFLP7) have been consistently recognized by various molecular techniques, including PCR-fingerprinting [6,7], restriction fragment length polymorphism (RFLP) analysis [8], amplified fragment length polymorphism (AFLP) analysis [9] and multilocus sequence typing (MLST) [10]. More recently, a fifth AFLP type (AFLP10) was described based on a single isolate [11]. These molecular types have been proposed for some time to be recognized as either varieties [12] or, more recently, as distinct species [13]. However, for the purpose of the current study they will be treated as distinct molecular types. Among *C*. *gattii*, the molecular type VGII, serotype B, has caused most of the outbreak-related cases, via clonal dispersion of three sub-genotypes, VGIIa, VGIIb and VGIIc [14–17]. Independent cases of infections caused by the molecular types VGI, VGIII and VGIV have been reported less frequently. VGI is the most prevalent molecular type in Australia and Papua New Guinea, where it is considered endemic [18,19]. VGIII is found predominantly in clinical and environmental sources in Mexico, Colombia and the USA [20–24], and VGIV has been mainly reported from India, African and a number of South American countries [8,25,26].

The number of *C*. *gattii* infections due to strains of the major molecular type VGIII is not only increasing in the endemic areas in South and Central America but also in the USA, where it is now a major cause of human and animal disease, with increasing cases reported from the Southeastern parts, especially California [17,27–29]. A total of 42 cases of human disease alone have been recorded since 2010, with the obtained genotypes being highly similar to the ones seen in both veterinary and environmental isolates. In contrast to the above-mentioned clonal VGII population, VGIII isolates have been highly diverse as shown by MLST analysis. This diversity has been attributed to the existence of both mating types α and **a** amongst clinical, veterinary and environmental VGIII isolates, which provides indirect evidence of sexual reproduction and dynamic recombination [17,27]. Furthermore, in South America and India, *C*. *gattii* VGIII has been isolated from the environment, although a specific association between its occurrence in the environment in these countries and cases of cryptococcosis has yet to be explored [22,30,31]. In a recent study, however, VGIII isolates recovered from arboreal sources in Southern California were genetically related to those causing human cases in similar locations, suggesting that these environmental isolates were the source of human infections [32].

Besides the *C*. *gattii* VGIII infections reported amongst HIV positive patients from Southern California and Mexico [20,21,33], the same molecular type has been recovered from immunocompetent human and veterinary patients. Several cases, including fatal infections, have been reported in patients without predisposing risk factors in Brazil, Colombia, Cuba, Mexico and the USA [17,23,24,34–39]. The emergence of *C*. *gattii* infections in immunocompetent patients is a source of public health concern as cryptococcosis is not usually suspected in this group and significant delays in diagnosis are associated with adverse outcomes. In apparently healthy hosts, cryptococcosis typically presents with cerebral involvement and is associated with more severe neurological sequelae, such as stroke, blindness, deafness and permanent neural deficits [37,39–41].

In addition, *C*. *gattii* VGIII isolates have been reported to be more susceptible to amphotericin B and 5-flucytosine than isolates of *C*. *neoformans* and other *C*. *gattii* molecular types [42,43]. Although azoles may have good *in vitro* activity against VGIII isolates, veterinary isolates of molecular type VGIII exhibited a wide range of minimum inhibitory concentrations (MICs) for fluconazole, with MICs as high as 32 μg/ml [28]. Previous studies have also indicated that VGIII is the most virulent molecular type in a *Drosophila* model of infection [44].

These differences have brought about the need to evaluate the epidemiology, disease transmission and virulence factors of this emerging pathogen. Therefore, the current study aimed to characterize *C*. *gattii* VGIII isolates genetically and phenotypically. In particular, to investigate the population genetic structure of VGIII isolates and to correlate geographic origin, source (clinical, veterinary or environmental), virulence in a mouse model of infection, and antifungal susceptibility. The data obtained from these investigations established a clearer understanding of the epidemiology and pathogenicity of *C*. *gattii* VGIII, revealed distinct ancestral populations, and found that there is no specific link between virulence and genotype of the studied isolates on the whole or mitochondrial genome level.

## Methods

### Isolates

One hundred twenty-two isolates of *C*. *gattii* molecular type VGIII from Australia (n = 1), Colombia (n = 37), Guatemala (n = 1), Mexico (n = 14), New Zealand (n = 1), Paraguay (n = 1), the USA (n = 66) and Venezuela (n = 1), were studied. Isolates were stored at -80°C in glycerol in the Westmead Millennium Institute Culture Collection (Australian Medical Mycology Culture Collection) located at the Molecular Mycology Research Laboratory, Westmead Millennium Institute, The University of Sydney, Sydney Medical School, Sydney, Australia (S1 Table). Among these isolates 56 were clinical, 38 veterinary and 28 of environmental origin. Standard strains of the four major molecular types of *C*. *gattii*, WM 179 (VGI/AFLP4, serotype B), WM 178 (VGII/AFLP6, serotype B), WM 175 (VGIII/AFLP5, serotype B) and WM 779 (VGIV/AFLP7, serotype C) were included as reference strains for the genotypic analysis [10]. The proposed type culture of the AFLP10 strain [13] was not included, as it was not publicly available at the time this study was undertaken.

Isolates were cultured on Sabouraud dextrose agar and incubated for 48 h at 27°C prior to DNA extraction. Genomic DNA was extracted as described previously [45].

### Molecular type, mating type and serotype identification

Restriction fragment length polymorphism (RFLP) analysis of the orotidine monophosphate pyrophosphorylase gene (*URA5*) following double digestion with the enzymes *Sau*96I and *Hha*I (New England BioLabs Inc) was used to identify the major molecular type of the isolates as reported previously [8].

Mating type was determined by PCR using the primers MfαU and MfαL for mating type α and MF**a**2U and MF**a**2L for mating type **a** and the PCR and amplification conditions as described previously [46].

The serotype of all isolates was determined using RFLP analysis of the capsular polysaccharide gene (*CAP59*), digested with the enzyme *Age*I (New England BioLabs Inc), as described previously [47]. The serotype of 53 selected isolates (23 of serotype B and 30 of serotype C) was in addition determined using the agglutination test CryptoChek (Iatron Laboratories, Tokyo, Japan) according to the manufacturer’s instructions (S1 Table). In all instances, serotypes were concordant by both methods.

Sequences of 360 bp of the partial region of the *CAP59* gene, which is used to determine serotype by RFLP, were extracted from the isolates with Whole Genome Sequencing (WGS) and a maximum likelihood dendrogram of these sequences was constructed to show the clear separation between serotypes B and C isolates (S1 Fig). The alignment and dendrogram were generated using the program MEGA 6.0 [48].

### Multilocus sequence typing (MLST)

MLST typing was performed using the International Society of Human and Animal Mycology (ISHAM) consensus MLST scheme for *C*. *neoformans* and *C*. *gattii*, which includes seven genetic loci, *CAP59*, *GPD1*, *LAC1*, *PLB1*, *SOD1*, and *URA5* genes, and the IGS1 region, as described previously [10]. All loci were amplified independently and the obtained PCR products were purified and commercially sequenced by Macrogen Inc., Seoul, Korea. Sequences were edited and contigs were assembled using Sequencher 5.3 (Gene Codes Corporation, Ann Arbor, USA). Each unique sequence was assigned an allele type (AT) number and the seven allele types per strain were subsequently combined to give a unique sequence type (ST) according to the ISHAM consensus MLST database, accessible at http://mlst.mycologylab.org. Alignments were generated using the program MEGA 6.0 [48]. The dendrogram showing the genetic relationship between the isolates based on the maximum likelihood analysis of the seven concatenated loci was generated using the same program. Haplotype network analyses were performed using the software Network 4.6.1.3 (Fluxus Technologies Ltd., Suffolk, UK). The goeBURST algorithm using the PHILOVIZ software (http://www.phyloviz.net) was used to generate a minimum spanning tree of the concatenated sequences to visualize relatedness of the *C*. *gattii* isolates according to the source of isolation and serotype. The diagrams show when the STs differ in a single locus variant (SLV), double locus variant (DLV), and triple locus variant (TLV). A clonal complex (CC) concept was adopted when SLV linkage with the founder ST was present [49].

### Genotypic diversity of the MLST loci

To estimate the genetic diversity amongst the STs, Simpsons diversity index (*D*) was calculated for the whole population, as well as by country, source, serotype and mating type of the isolates.

The length of each MLST locus, the number of alleles and their frequency were determined and the genetic diversity of the seven loci was estimated by calculating the average number of nucleotide diversity (π) and the number of polymorphic (segregating) sites (S) using the software DnaSP ver. 5.10.1 [50]. For comparison of the genetic diversity between the VGIII sub-populations, the index θ, which is the Weir's formulation of Wright's fixation index (F_ST_) for population differentiation analysis, was calculated for each locus. F_ST_ values of >0.05 generally indicate little inter-population variance, and can range from 0 for identical populations to 1 for populations with no alleles in common. The Index of Association (*I*_*A*_) and rBarD were also calculated to test for recombination between and within the VGIII sub-populations. Since clonal reproduction can mask the effects of recombination, *I*_*A*_ and rBarD were calculated using the clone-corrected data for each ST after removal of identical genotypes were removed (haplotypes only). The values of both *I*_*A*_ and rBarD are expected to be zero if populations are freely recombining and greater than zero if there is association between alleles. The rBarD statistic takes into consideration the number of loci tested and is considered a more robust measure of association. Values of F_ST_, *I*_*A*_ and rBarD were calculated using the program MultiLocus 1.3 [51].

### Coalescence analysis

The BEAST v1.8.3 software was used to perform the Bayesian molecular clock analysis of the VGIII MLST sequences [52]. The Tamura Nei model with invariable sites and gamma distribution (TrNef + I + G) used in BEAST was the best model selected from the Bayesian Information Criterion in the software jModelTest 2.1.7 [53]. The stepping-stone sampling marginal likelihood estimator available in MrBayes v3.2 software was used to infer the best-fitting clock model for the dataset [54].

A relaxed lognormal clock was applied to infer the time scale incorporating one internal node calibration of 8.5 million years as the time to most recent common ancestor for VGIII as already described [12]. A normal prior age distribution of 0.25 million years was used in the analysis. The XML file was generated in BEAUTI v1.8.3 with a run of 10^8^ generations, 1 tree sampled per 1,000 generations, and a burn-in of 10% [52]. The LogCombiner v1.8.3, distributed with BEAST, was used to combine the files of two independent runs applying a burn-in of 10%. The results were visualized using the Trace v1.6.0 software distributed with BEAST and showed that the effective sample size was higher than 200 in all analyses. The tree with the highest log clade credibility was selected in the software TreeAnotator v1.8.3 and the tree presenting the posterior mean and 95% confidence intervals of the time to most recent common ancestor was visualized in the FigTree v1.4.3 software (http://tree.bio.ed.ac.uk/software/figtree/).

### Whole genome sequencing (WGS)

Sixty isolates, comprising 33 serotype B and 27 serotype C, representing the full diversity of the VGIII MLST genotypes, were selected for WGS. High quality DNA was extracted with the ZR Fungal/Bacterial DNA MiniPrep kit (Catalog N° D6005; Zymo Research, Irvine, CA, USA) following the instructions of the manufacturer.

The samples were sequenced using Illumina HiSeq as previously described [16,55]. DNA samples were prepared for paired-end Illumina sequencing using the Kapa Biosystems Library Preparation with Standard PCR kit (Catalog N° KK8232; Woburn, MA, USA) protocol. Approximately 1μg of double-stranded DNA (dsDNA) was sheared using a Sonicman sonicator (Brooks automation, Spokane, WA, USA) to an average size of 650 bp and DNA libraries were prepared for sequencing as described by the manufacturer. Modified oligonucleotides (Integrated DNA Technologies, Coralville, IA, USA) with 8bp indexing capability [56] were substituted at the appropriate step. Libraries were quantified prior to sequencing with quantitative PCR (qPCR) on the ABI 7900HT (Life Technologies Corporation, Carlsbad, CA, USA) using the Kapa library quantification kit (Catalog N° KK4835; Woburn, MA, USA). Libraries were sequenced to a read length of 100bp on the Illumina HiSeq system. WGS read files were deposited in the NCBI Sequence Read Archive under BioProject PRJNA289249.

All sequenced samples were assembled *de novo* using the SPAdes v2.5.0 assembler [57]. Read data for all genomes were aligned against the *de novo* assembly for sample WM 175 using Novoalign 3.00.03 (Novocraft Technologies, Selangor, Malaysia). Single nucleotide polymorphisms (SNPs) were detected using the Genome Analysis Toolkit v2.4 (GATK) [58]. SNP calls were filtered using NASP (http://tgennorth.github.io/NASP/) and had to meet the following criteria per SNP loci to be included in the final matrix: coverage of a minimum 10X and less than 10% variant allele calls. Additionally, reads that mapped to multiple locations within the genome were excluded from the analysis, as were positions located in an insertion or deletion site. The *de novo* assembly of sample WM 1814 was used as the reference strain for serotype C analyses; otherwise WM 175 was used as the reference strain. Additionally, one isolate of each of the other three *C*. *gattii* major molecular types (VGI, VGII, and VGIV), previously sequenced by WGS, were included for phylogenetic analysis [15]. In total, three SNP matrices with different taxa were produced: (i) *C*. *gattii* molecular types VGI to VGIV; (ii) *C*. *gattii* molecular type VGIII only, and (iii) *C*. *gattii* major sub-populations (serotype B and C) together.

Whole-genome SNP typing (WGST) was performed as previously described [16,59,60] for phylogenetic analysis in order to understand genetic relationships between isolates. To put the VGIII population in context with the other *C*. *gattii* major molecular types the genomes of the following *C*. *gattii* isolates were retrieved from GenBank: WM 179 and WM 276 representing molecular type VGI, WM 178, WM 05.419, WM 04.78, WM 06.12, WM 08.309, CDCR265, CDCR272, B9816 and GT 11.7650 representing molecular type VGII, and WM 779 representing molecular type VGIV [15,60]. Maximum parsimony SNP trees were constructed using PAUP* v.4.0b10 (Sinauer Associates, Inc., Sunderland, MA, USA) and visualized using FigTree v.1.3.1 (http://tree.bio.ed.ac.uk/software/figtree/). The *C*. *gattii* VGI to VGIV tree was not rooted. Additionally, the SNP matrix (iii) was examined for recombination using the phi test [61]. The neighbor-joining split tree network was drawn on the SNP matrix (iii) in order to visualize the existing recombination between samples using the program SplitsTree4 [62,63] with the uncorrected P-distance transformation. A maximum likelihood tree with 1,000 bootstrap generations was produced from SNP matrix (ii) using the TVM+ASC+G4 model in IQ-TREEv1.3.10 [64]. The tree was visualized using FigTree v1.3.1.

*fineStrucutre* analysis [65] was performed on the SNP matrix (ii) and (iii) in order to infer the population structure within VGIII as well as identify admixture events occurring between molecular types. Using the phylogenetic tree produced above, one representative from each clonal clade was selected and the SNP matrix was reduced to a pairwise similarity matrix using Chromopainter, which was run using the linkage model and assuming uniform rates of recombination per base pair of sequence. Populations were determined through *fineStructure* using the above-mentioned similarity matrix.

Putative gene content comparison was performed using BLAST score ratio (BSR) analysis [66], as previously described [15]. VGIII serotype B and serotype C predicted gene content differences were confirmed by alignment of sequence read data. Putative gene characterization of confirmed gene differences were translated into amino acid sequences and searched against the Pfam database (http://pfam.xfam.org/) in order to identify potential protein functions. Additional characterization for selected putative genes were searched against the NCBI non-redundant protein database (http://www.ncbi.nlm.nih.gov/RefSeq/) using blastp.

### Mitochondrial genome analysis

In order to assess mitochondrial re-arrangements and mutations, four high-virulence and four low-virulence serotype B samples were assembled using SPAdes3.0 [67]. Mitochondrial genes of the highly virulent VGII CDCR265 strain from the Broad institute were used as reference sequences. Fifteen mitochondrial genes were used to identify the mitochondrial contigs in the eight genome assemblies using BLAT. Contigs containing the 15 mitochondrial genes were pulled from the assemblies and were aligned using progressiveMauve v2.3.1 [68].

Additionally, the 56kb contig containing the 15 mitochondrial genes from the isolate WM09.47 was used as a reference sequence for SNP analysis using NASP as described above.

### Virulence study

Based on the MLST results, 17 isolates were chosen to study the pathogenic potential of *C*. *gattii* molecular type VGIII in a murine model of pulmonary cryptococcosis. Female BALB/c mice, 6-weeks-old and weighing between 16 to 18 g, were inoculated intranasally with 10^5^ yeast cells suspended in sterile saline. Prior to inoculation, the isolates were grown in Sabouraud dextrose agar at 37°C for 24 hours. Five mice per cryptococcal isolate studied were weighed and anesthetized intraperitoneally by injecting 0.03 to 0.04 ml of a combination of ketamine (44 mg/ml) and midazolam (2.7 mg/ml) (80 mg of ketamine and 5 mg of midazolam in a total volume of 1.8 ml), using an insulin syringe. Following induction of anesthesia, the mice were hung on a silk thread by their incisor teeth, so that the necks were fully extended. By using a pipette, 50 μl of inoculum was slowly instilled directly into each nostril. The well-studied *C*. *gattii* strains CDCR265 (VGIIa, highly virulent) and CDCR272 (VGIIb, low virulence) from the Vancouver Island outbreak were included as reference strains for comparison [69]. Five mice were also inoculated with sterile saline as an inoculation control. After inoculation, mice were placed in standard cages with access to water and food *ad libitum* and weighed and observed daily for signs of infection (e.g. difficulty breathing, neurological signs, ruffled fur, lethargy, poor appetite), until the end point of 60 days. Affected mice were euthanized by CO_2_ (5%) inhalation immediately upon observation of any signs of distress. Necropsy was performed and the brain and lungs were collected for macroscopic and histopathological examinations, to determine the presence of yeast and lesions. Blood collected aseptically from the heart using an insulin syringe was plated on Sabouraud dextrose agar to check for hematogenous dissemination. After harvesting tissues at necropsy, infected material was autoclaved and disposed of by incineration.

To compare the virulence of selected isolates, survival curves for each isolate were graphed. Median survival times were obtained and differences in survival times were analyzed by the Log-rank (Mantel-Cox) test. Statistical analysis and plots were carried out using GraphPad Prism version 6.0b (La Jolla, CA, USA). In all cases, *p*-values of <0.05 was considered statistically significant.

### Antifungal susceptibility

Susceptibility testing was carried out using the Sensititre YeastOne plate (Thermo Scientific, USA), which is a colorimetric microdilution test, following the manufacturer’s instructions. Briefly, isolates were grown on Sabouraud dextrose agar and incubated for 24 h at 27°C. Discreet yeast colonies were suspended with a swab into 5 ml of sterile water, adjusted to a density of 0.5 McFarland standard (1–5 × 10^6^ cells/ml), and 20 μl aliquots were transferred into 11 ml of YeastOne inoculum broth for a final concentration of 1.5–8 × 10^3^ CFU/ml. An aliquot of 100 μl of inoculum was placed in each well of the Sensititre YeastOne plate using a multichannel pipette. Plates were sealed, incubated at 35°C and read manually after 72 h of incubation.

The reference strains of *Candida krusei* ATCC 6258 and *Candida parapsilosis* ATCC 22019, were used as quality control. Controls were read after 24 h of incubation.

Purity of the cell suspension and colony counts were determined by plating 10 μl of inocula on to Sabouraud dextrose agar. The range of drug concentrations tested by 2-fold serial dilutions was 0.125–8 μg/ml for amphotericin B; 0.125–256 μg/ml for fluconazole; 0.015–16 μg/ml for itraconazole; 0.008–8 μg/ml for voriconazole and posaconazole; and 0.06–64 μg/ml for 5-flucytosine.

The range of minimum inhibitory concentrations (MICs), MIC_50_, MIC_90_ and geometric mean MICs of each antifungal drug were estimated. Epidemiologic cutoff values (ECV), defined as the MIC value encompassing at least 95% of the wild-type distribution, were calculated for each antifungal drug. Significant differences in MICs between two groups of isolates were compared using a Mann-Whitney test. Group comparisons for MIC data included serotype B *vs*. serotype C; mating type α *vs*. mating type **a**; clinical *vs*. veterinary isolates; clinical *vs*. environmental isolates and veterinary *vs*. environmental isolates. All analyses were performed with GraphPad Prism version 6.0b (La Jolla, CA, USA); *p*-values <0.05 were considered significant.

### Ethics statement

The virulence study was carried out in accordance with the protocol No. 4151-06-09 approved by the Westmead Hospital Animal Ethics Committee (WHAEC) adhering to Australian Code for the Care and Use of Animals for Scientific Purposes 8^th^ Edition 2013 and the Animal Research Act New South Wales 1995.

## Results

### The *C*. *gattii* VGIII population is represented by serotypes B and C isolates with mating types α and a identified in both serotypes

RFLP analysis of the *URA5* gene identified 116 of the 122 isolates as molecular type VGIII and six isolates displayed the restriction pattern of the molecular type VGIV. *In silico* restriction and alignments of the *URA5* sequences of these six isolates, revealed a single nucleotide polymorphism (SNP) in the position 528, which is the restriction site of the enzyme *Sau*96I, resulting in misidentification of those isolates as VGIV [12]. However, as MLST analysis established that all 122 isolates were related, they were classified as molecular type VGIII.

Serotype and mating type analysis identified 60 (49%) isolates as B/*α*, 39 (32%) as C/*α*, 15 (12%) as B/**a** and 8 (7%) as C/**a**. The obtained serotype data were confirmed based on *CAP59* sequences extracted from the whole genome sequencing (WGS) data (S1 Fig). Detailed descriptions of the mating and serotype results obtained for each isolate are provided in S1 Table.

### MLST analysis revealed two major clades among the *C*. *gattii* VGIII isolates and linked clinical and veterinary cases with environmental sources

Among the 122 isolates, 55 sequence types (STs) were identified (S1 Table). Of these, ST75 was the most frequent sequence type (21 serotype B isolates: five clinical and 15 veterinary isolates from the USA and one clinical isolate from Mexico), followed by ST79 (16 serotype C isolates: four clinical and nine environmental isolates from Colombia, two clinical isolates from Mexico and one from the USA). ST116 was the third most common sequence type, containing seven serotype B environmental isolates from Colombia.

Of the remaining 52 STs, 37 were represented by a single isolate each, while 15 were represented by two to five isolates, with ST65 (n = 2) and ST68 (n = 2), ST146 (n = 3) and ST74 (n = 5), each identified in more than one country. ST65, ST146 and ST74 each contained isolates from different source (S1 Table).

**Fig 1:**
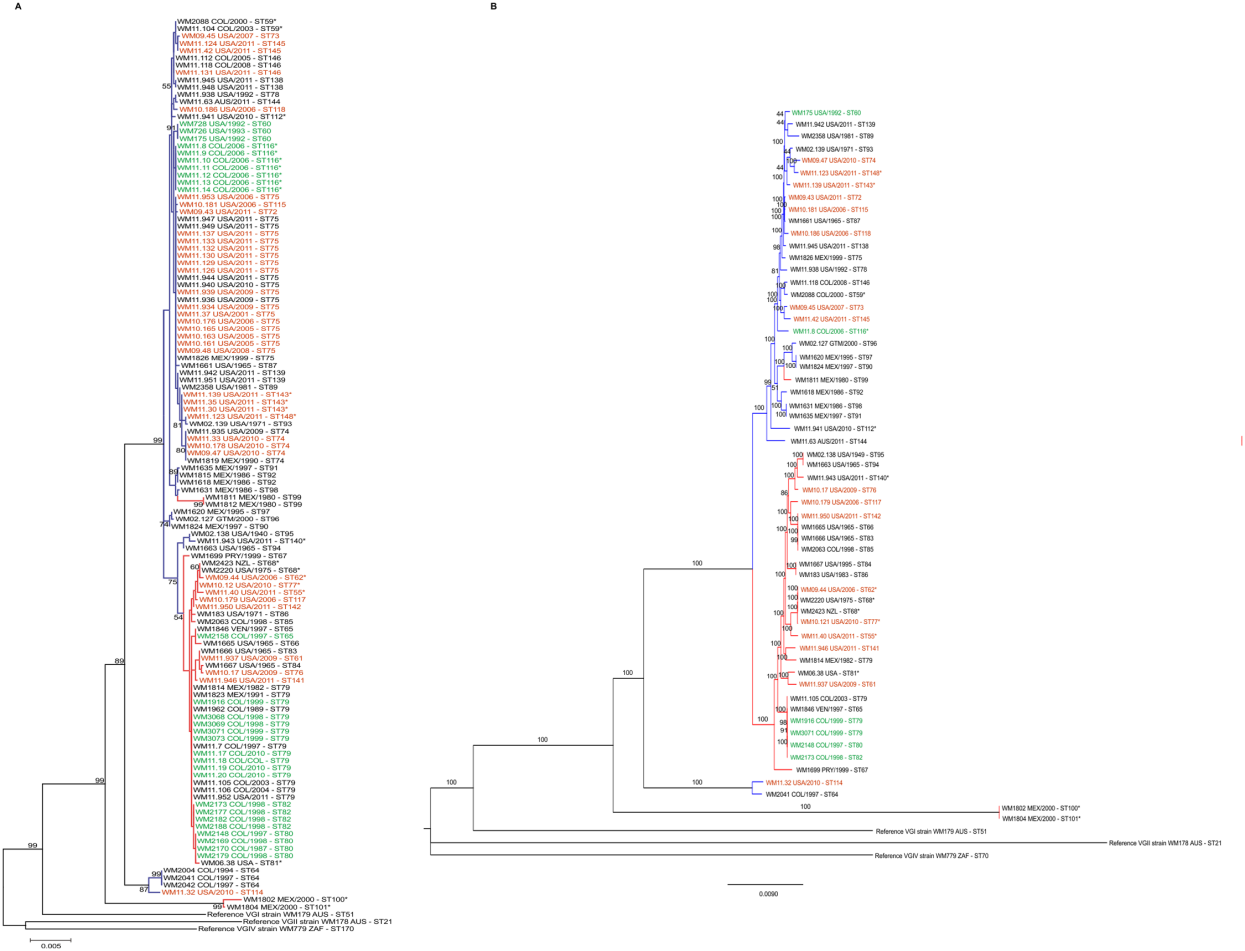
**Dendrogram showing the genetic relationships between 122 *Cryptococcus gattii* molecular type VGIII isolates** based on the analysis of the seven concatenated MLST loci of the International Society for Human and Animal Mycology (ISHAM) consensus MLST scheme, using the program MEGA 6.0 [48] **(A). Dendrogram showing the genetic relationships between 60 *C*. *gattii* isolates representing all published MLST sequence types for molecular type VGIII currently present in the MLST database based on the WGS SNP matrix (ii) (B).** Numbers on the branches indicate bootstrap values above 50. The color of the branches indicates serotype B (blue) and C (red). Color of the font indicates clinical (black), veterinary (orange) and environmental (green) isolates recovered in AUS: Australia, COL: Colombia, GTM: Guatemala, MEX: Mexico, NZL: New Zealand, PRY: Paraguay, USA: USA, VEN: Venezuela and ZAF: South Africa (Country abbreviations according to the United Nation (UN) three letters ISO 3166–1 code). Asterisk (*) indicates mating type **a** isolates, all other isolates are mating type *α*.

**Fig 2:**
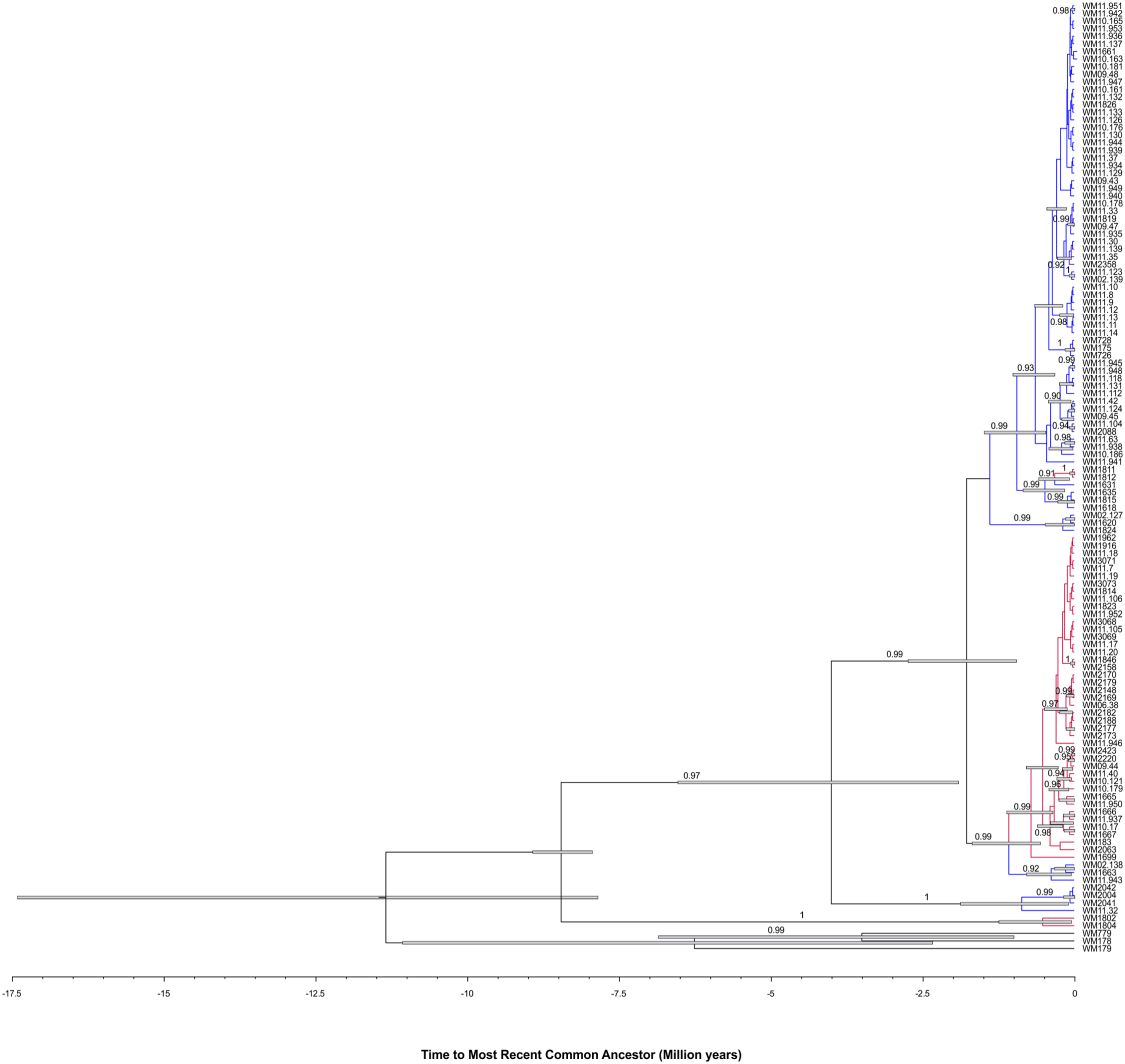
Coalescence gene genealogy of the *Cryptococcus gattii* isolates evidencing the presence of two main clusters (Serotypes B and C) within the VGIII genotype that separated 0.96 to 2.7 million years ago. Blue bars represent the 95% high posterior probability of the ages in the branches with a posterior probability limit of 0.5. The Bayesian posterior support values higher than 90% are described in the branches of the tree. The serotype C isolates are highlighted in red while serotype B isolates are highlighted in blue. The reference strains WM 179 (VGI, ST51), WM178 (VGII, ST21), and WM779 (VGIV, ST70) were included in the analysis as out-groups.

Based on maximum likelihood analysis and coalescence analysis of the seven concatenated MLST loci, *C*. *gattii* molecular type VGIII isolates separated into two major clusters or sub-populations corresponding mainly to serotype B and C (Figs 1A, 2, S1 Fig). These two sub-populations most likely correlate with the VGIIIa and VGIIIb lineages, respectively, that were recently described in independent MLST studies using a different MLST scheme [20,32], based on the loci that both MLST schemes share (*GPD1*, *LAC1*, *PLB1* and IGS1) [10,20,32].

Among the serotype B isolates, WM 1811 and WM 1812 (ST 99), were identified as serotype C. However, because they shared most of the MLST alleles with the serotype B isolates, they were considered as such for the purpose of the analysis. Similarly, isolates WM 02.138 (ST95), WM 11.943 (ST140) and WM 1663 (ST94) were identified as serotype B, but were considered as serotype C for the analyses, as they clustered more closely with isolates of the later serotype.

Isolates outside the two major clusters, namely the six isolates misidentified as VGIV by *URA5*-RFLP, mentioned previously, grouped into two additional small sub-populations, with each corresponding either to the serotype B (n = 4) or C (n = 2) (Figs 1A, 1B and 2). From these atypical strains, we deduced the presence of a novel serotype B, VGIII ancient lineage among *C*. *gattii* VGIII isolates, represented by three isolates from Colombia (WM 2004, WM 2041 and WM 2042 (ST64)) and one isolate from the USA (WM 11.32 (ST114)), reported herein for the first time. The coalescence analysis showed that these isolates diverged from the VGIII isolates around 1.91 to 6.53 million years ago. A second ancient lineage that diverged from VGIII isolates around 7.94 to 8.92 million years ago, represented by two serotype C isolates (WM 1802 (ST100) and WM 1804 (ST101)) from Mexico, likely corresponds to the previously described VGIIIc/AFLP10 lineage [11,32], as these isolates share most of their MLST alleles with the published strain CBS11687 (IHEM14941 = RV 63979), with the exception of the *SOD1* allele [11,13]. Both lineages appear to be basal to the VGIII clade (Figs 1A, 1B and 2).

The sequences obtained for each allele type were deposited in GenBank under the following accession numbers: *CAP59* (JX840782—JX840787), *GPD1* (JX840788—JX840795), *LAC1* (JX840805—JX840821), *PLB1* (JX840822—JX840832), *SOD1* (JX840833—JX840840), *URA5* (JX840841—JX840851), and IGS1 (JX840796—JX840804) (S2 Table or at the INTERNATIONAL FUNGAL MLST DATABASE website at mlst.mycologylab.org).

### Genotypic diversity of the MLST loci shows low genetic flow between serotypes B and C but some degree of recombination within each

The VGIII population was genetically highly diverse (*D* = 0.061). There were no statistically significant differences among the groups with respect to the country of origin, isolate source (clinical *vs*. veterinary *vs*. environmental), or mating type. Only minor differences were identified between serotypes, with serotype C isolates being slightly more diverse than those of serotype B (*D* = 0.056 *vs*. 0.105 (*p* < 0.05)) (S3 Table).

Among the seven loci studied, *LAC1* was the most informative locus with 17 alleles, 31 polymorphic sites over 477 bp, a nucleotide diversity of 0.895%, and a fixation index (F_ST_ or θ) of 0.5137. Although *SOD1* was represented by eight alleles, two more than *CAP59*, it was the least informative locus with only 16 polymorphic sites over 713 bp and a nucleotide diversity of 0.205% (Table 1). Overall, the seven concatenated loci resulted in an alignment of 4,212 bp with 160 polymorphic sites. The high F_ST_ values of all seven loci indicate high genetic diversity between the VGIII sub-populations (serotype B and serotype C clusters, respectively), indicating low genetic flow between them (Table 1). The low number of shared MLST alleles between serotype B and serotype C isolates (S1 Table), and the high values obtained with the tests of linkage disequilibrium (*I*_*A*_ = 1.16184 and rBarD = 0.197630 (*p* = 1.00)), further support low genetic flow within this population structure. Tests of linkage disequilibrium showed some recombination within each sub-population, with serotype B isolates (*I*_*A*_ = 0.478372 and rBarD = 0.0962771 (*p* < 0.01)) recombining less frequently than serotype C isolates (*I*_*A*_ = 0.342210 and rBarD = 0.0575250 (*p* = 0.02)).

**Table 1:** Allele diversity per MLST locus of the studied *Cryptococcus gattii* molecular type VGIII isolates (n = 122).

Locus	Length (bp)	n of alleles	Nucleotide diversity (π)	n of polymorphic sites (S)	Fixation index F_ST_ (θ)
*CAP59*	557	6	0.534%	19	0.9513
*GPD1*	549	8	0.371%	18	0.6681
IGS1	743	9	0.372%	38	0.5500
*LAC1*	477	17	0.895%	31	0.5137
*PLB1*	535	11	0.331%	18	0.5290
*SOD1*	713	8	0.205%	16	0.5204
*URA5*	638	11	0.299%	20	0.5337

**Fig 3:**
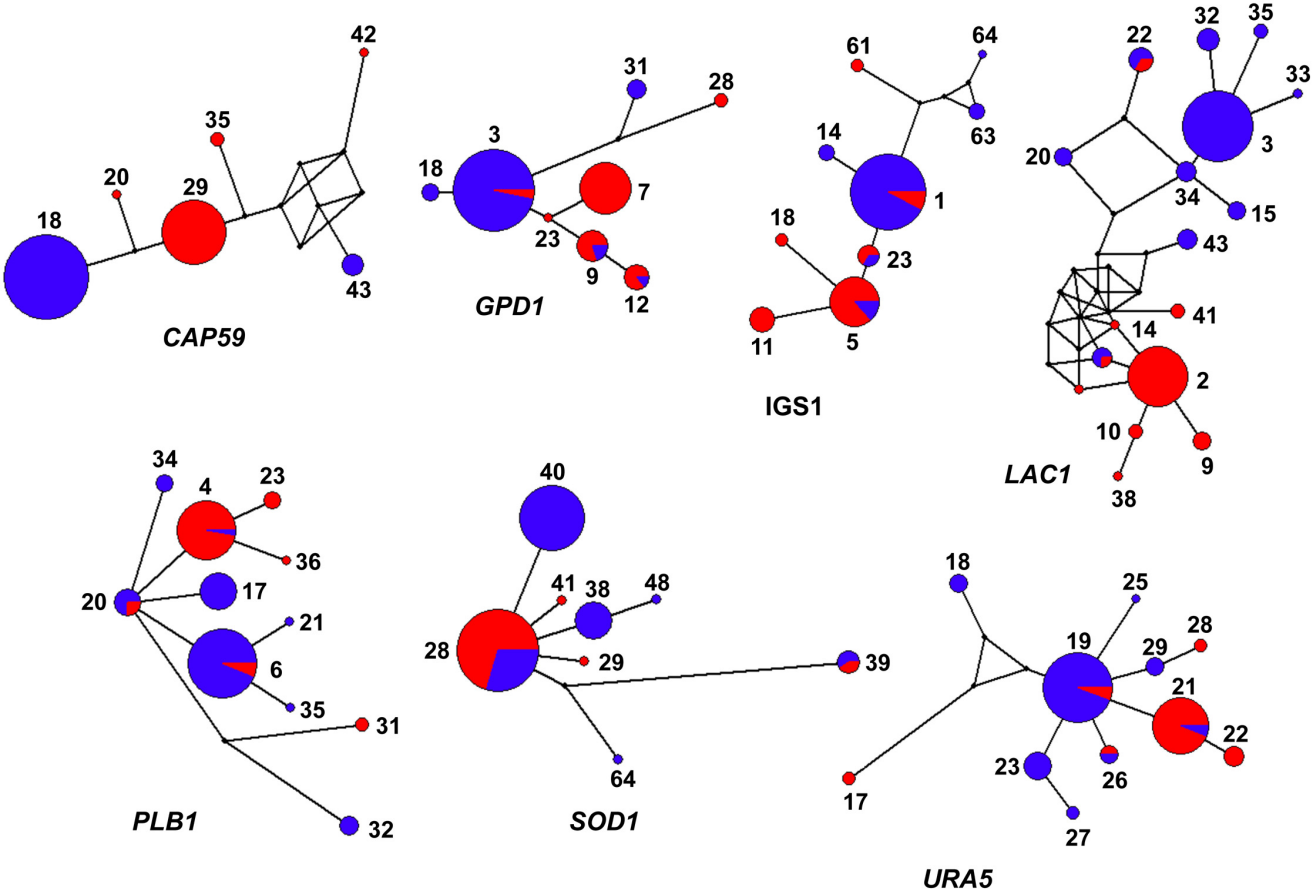
Haplotype network analysis by locus of the studied *Cryptococcus gattii* molecular type VGIII isolates. Networks were constructed using the software Network 4.6.1.3. The size of the circles is proportional to the number of isolates. Color of the circles indicates serotype B (blue) and serotype C (red). Dual-coloration represents shared alleles between serotypes. Alleles for each locus are indicated numerically.

**Fig 4:**
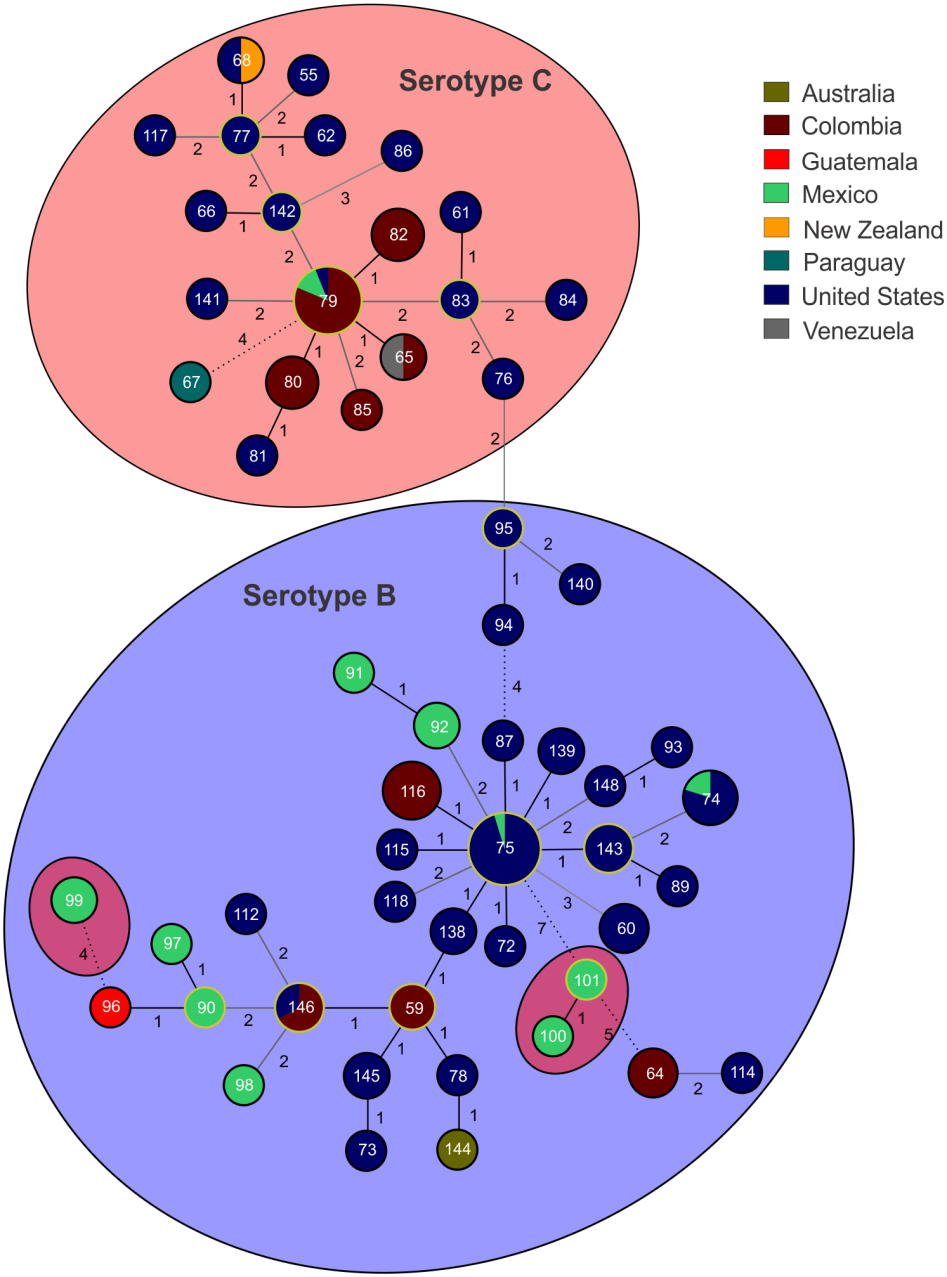
Minimum spanning tree of the studied *Cryptococcus gattii* molecular type VGIII isolates. The tree was constructed using the goeBURST algorithm and shows the distribution of the STs according to the country of origin and the serotype B (blue) or serotype C (red) status of the isolates. STs are indicated numerically inside the circles. The size of the circles is proportional to the number of isolates per each ST. Circles surrounded by yellow lines represent the founder of each clonal complex. Numbers between STs represent one (black solid line), two (dark grey line), three (light grey line), and more than four (dashed line) allele differences.

Haplotype network analyses per locus revealed a low number of shared MLST alleles between sub-populations, indicating a low level of recombination (Fig 3). The low number of shared alleles between these two *C*. *gattii* VGIII sub-populations was also supported by the goeBURST analysis with the concatenated dataset (Fig 4). Overall, only the four atypical serotype C isolates from Mexico (WM 1802 (ST100), WM 1804 (ST101), WM 1811 (ST99) and WM 1812 (ST99)), shared alleles, and were grouped in the serotype B cluster. This analysis also presented 11 clonal complexes (CC) (i.e. 11 groups presenting single locus variant (SLV)) with two of them, CC79 (composed of ST79, ST82, ST80, and ST65 in serotype C group) and CC75 (composed of ST75, ST115, ST138, ST72, ST143, ST139, and ST87 in the serotype B group), appearing to play an important role in the epidemiological distribution of the *C*. *gattii* VGIII population due to the wide geographical distribution of the CCs (Fig 4).

### Whole genome sequencing supports the presence of sub-populations among the *C*. *gattii* VGIII isolates

**Fig 5:**
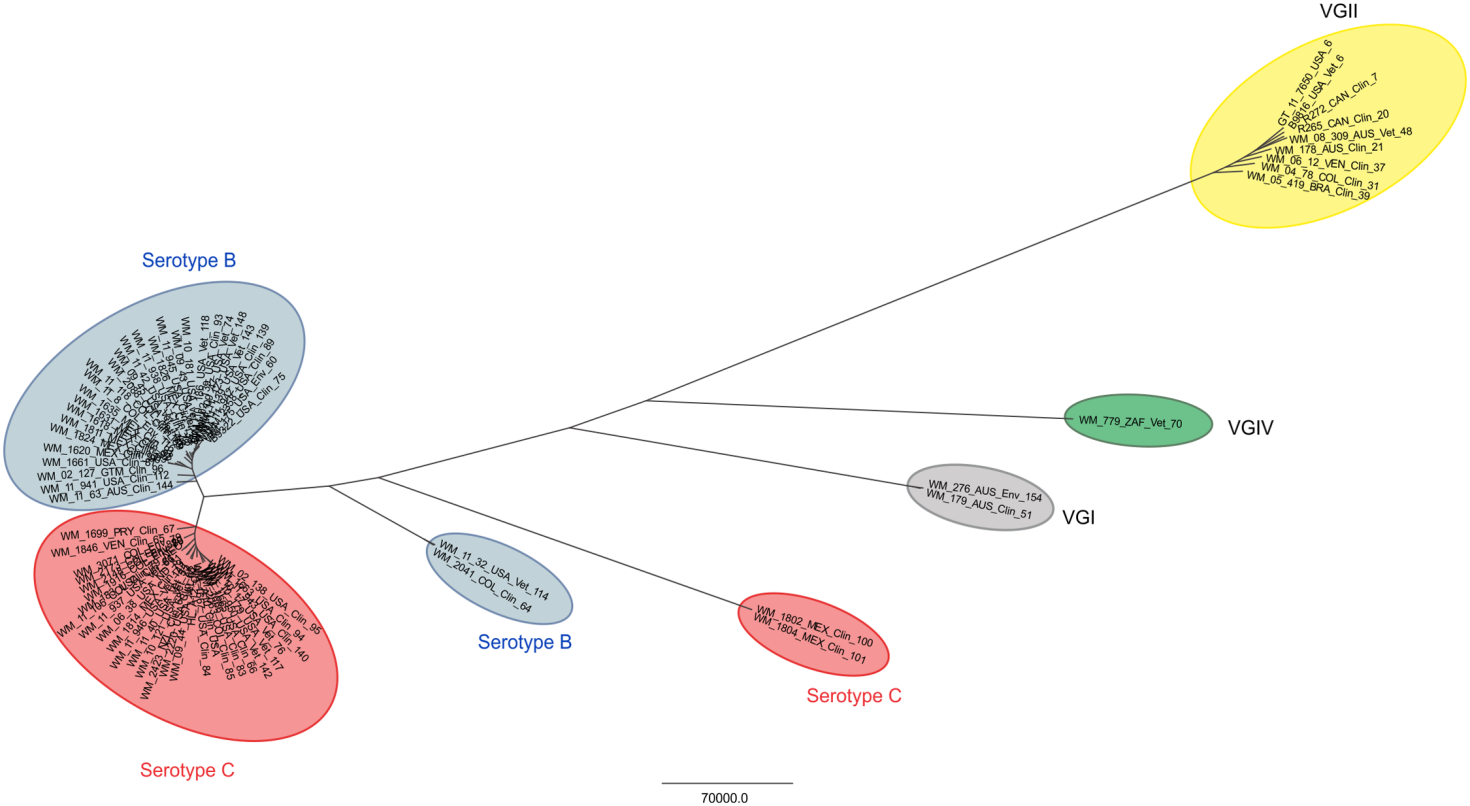
Maximum parsimony phylogenetic analysis of the major molecular types of *Cryptococcus gattii* (VGI to VGIV) performed on WGST SNP data. The analysis of the molecular type VGIII alone found 572,268 total SNPs, with 514,098 of them being parsimonious SNPs, with a consistency index (CI) of 0.7691. The tree shown is not rooted. Branch lengths represent numbers of SNPs between taxa, with the unit bar in the Figure. The taxa nomenclature include a unique strain identifier, the country and source of isolation and the ST. AUS: Australia, COL: Colombia, GTM: Guatemala, MEX: Mexico, NZL: New Zealand, PRY: Paraguay, USA: USA, VEN: Venezuela and ZAF: South Africa; Clin: clinical, Vet: veterinary, and Env: environmental.

Whole genomes from 60 *C*. *gattii* VGIII isolates, representing at least one isolate per ST identified in the MLST analysis, were sequenced. Whole genome sequencing (WGS) determined the presence of 572,268 SNPs with 514,098 SNPs being parsimony informative. Within the serotype B and serotype C major sub-populations, 88,337 and 79,945 SNPs were identified, respectively. Maximum parsimony analysis based on whole genome SNP typing (WGST) confirmed the same clustering of the VGIII isolates as obtained by MLST typing, although with much higher resolution (Figs 1B and 5). When the whole genomes of the other major molecular types of *C*. *gattii*, VGI, VGII, and VGIV [15] are included (Fig 5), WGST SNP data found 1,347,295 total SNPs with 1,055,552 of them being parsimonious SNPs, with a consistency index (CI) of 0.7934.

**Fig 6:**
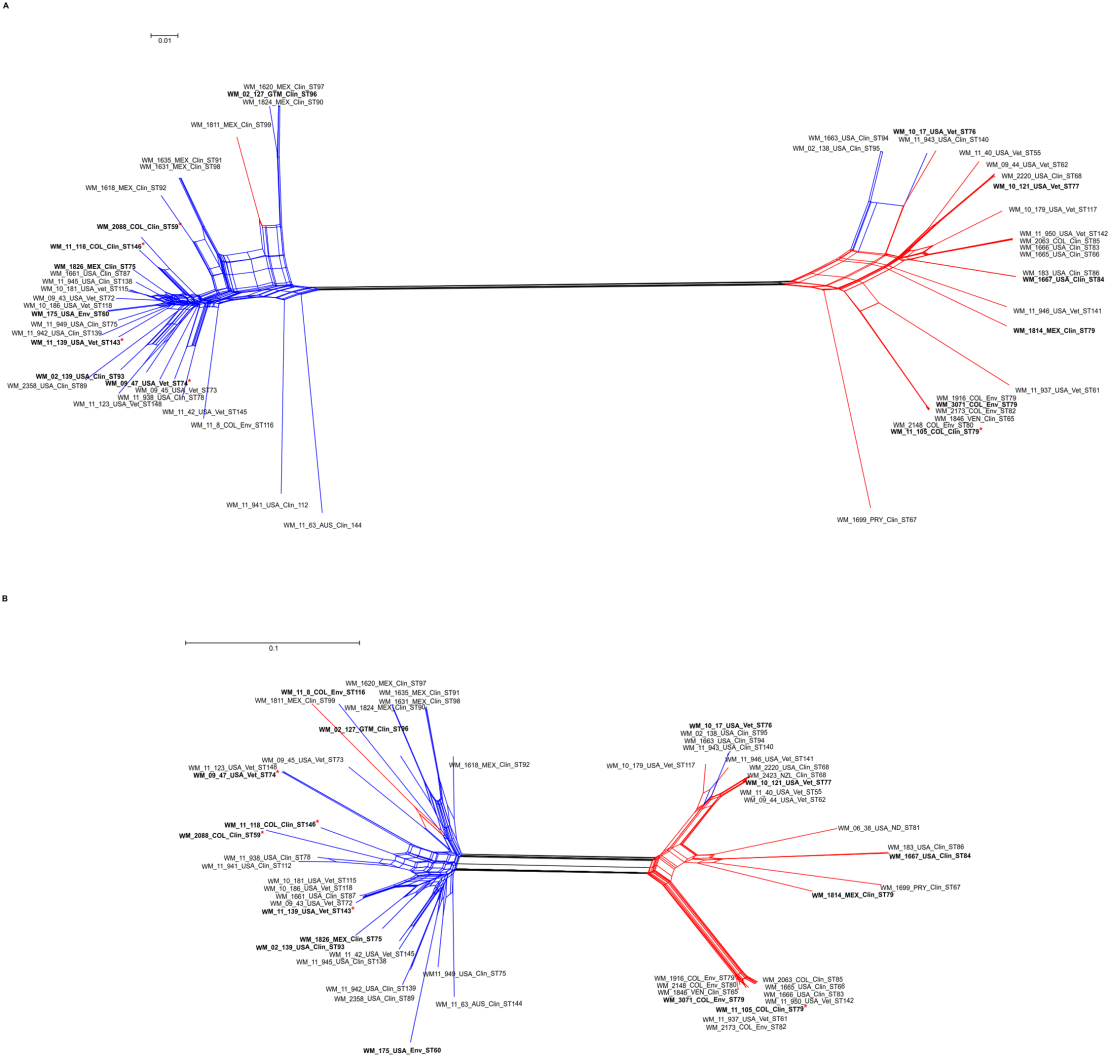
Phylogenetic network of the main sub-populations of *Cryptococcus gattii* molecular type VGIII based on whole genome sequence analysis (A) and mitochondrial genome analysis (B). The neighbor-joining phylogenetic splits tree represents the relationship between the VGIII STs and serotypes using the uncorrected P distance transformation. Each band of parallel edges indicates a split. The taxa nomenclature include a unique strain identifier, the country and source of isolation and the ST. AUS: Australia, COL: Colombia, GTM: Guatemala, MEX: Mexico, NZL: New Zealand, PRY: Paraguay, USA: USA, and VEN: Venezuela; Clin: clinical, Vet: veterinary, and Env: environmental. Isolates included in the virulence studies are in bold. High virulent strains are indicated with a red asterisk.

Neighbor-joining phylogenetic splits tree network analysis of the WGST SNP data clearly separated the major serotype B and serotype C clusters within VGIII and showed many phylogenetic splits within each sub-population (Fig 6A). These findings indicate a shared genetic history, possibly including sexual recombination events within, but not between the two main serotype groups in the VGIII population, which largely contributes to the genetic diversity found within each serotype. When the Phi test for recombination was performed using the SNP data, the test indicated that recombination was present within each of the two major VGIII serotype groups (p = 0.0).

**Fig 7:**
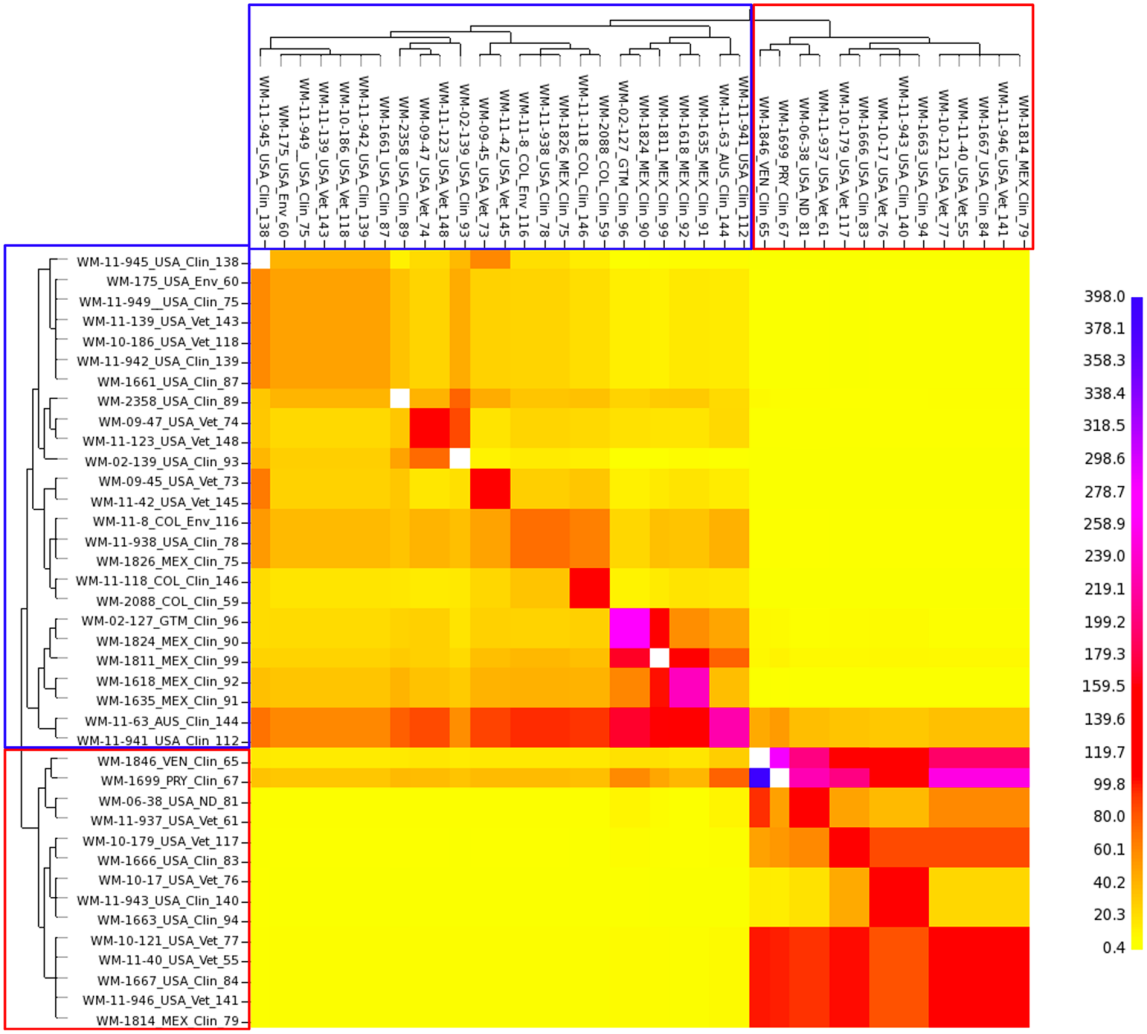
*fineStructure* analysis of the main sub-populations of *Cryptococcus gattii* molecular type VGIII. To identify the population structure and possible recombination events between and within serotype B and C, *fineStructure* analysis was performed using a SNP matrix (iii). Whole-genome SNP data were reduced to a pairwise similarity matrix. The x-axis represents the strain as a “donor” and the y-axis represents the strain as a “recipient” of genomic regions. The scale bar represents the number of shared genome regions with blue being the greatest amount of sharing and yellow being the least. Blue and red boxes represent serotype B and serotype C isolates, respectively.

*fineStructure* analysis showed that the serotype B isolates shared very few or no genomic regions with the serotype C isolates. However, within sub-populations, there are some shared genome regions, with serotype C isolates having a greater amount of shared regions than serotype B isolates, indicating a significant separation between the two major VGIII serotype groups, but at the same time also suggestive of some level of recombination within each of them (Fig 7). In addition *fineStructure* analysis indicates incomplete lineage sorting among the atypical strains, accounting for the maintenance of ancestral genome parts (S2 Fig). The VGI genome contributed more to the genomes of the atypical VGIII serotype B strains (WM 1802, WM 1804) and the VGIV genome contributed stronger to the genomes of the atypical VGIII serotype C strains (WM 2004, WM 2041, WM 2042 and WM 11.32) (S2 Fig). If these ancestral groups are isolated (genomically and geographically) they would have very little recombination opportunity and little new variation, and therefore do not “share” their genome with others.

Very few differences in gene content were found between serotype B and serotype C isolates using the BLAST Score Ratio (BSR). An analysis of the presence/absence of genes in the VGIII sub-populations identified two gene clusters that were unique to the serotype C genomes, and one gene cluster that was unique to the serotype B genomes. However, all three gene clusters represented hypothetical proteins of unknown function. Both clusters identified in the serotype C isolates, did not have significant matches (E values 7.00E-51 and 3.00E-109), and although the cluster identified in the serotype B isolates matched with a H-N-H homing endonuclease (E value 8.00E-79), the amino acid identity was only 50% with 96% coverage.

### Mitochondrial genome analysis supports two sub-populations amongst the VGIII isolates

Based on previous findings, implicating changes in mitochondrial morphology and mitochondrial gene expression to an increased virulence in the Vancouver Island Outbreak VGII strains [70], the mitochondrial genomes were bioinformatically extracted from the WGS data set of the 60 *C*. *gattii* VGIII isolates, representing at least one isolate of each ST identified in the above mentioned MLST analysis. Interestingly, the estimated mitochondrial genome size of *C*. *gattii* VGIII strains was 55 kb, which is much larger than the mitochondrial genome sizes of *C*. *gattii* VGII strains (34.7 kb) [70], *C*. *neoformans* var. *grubii* strains (24 kb) and *C*. *neoformans* var. *neoformans* strains (32 kb) [71]. Mitochondrial genome sequencing determined the presence of 577 SNPs with 415 SNPs being parsimony informative, with a consistency index (CI) of 0.36. Neighbor joining phylogenetic splits tree network analysis of the mitochondrial genomes confirmed a similar but not identical topology for the two major VGIII clusters identified in the WGS analysis (Fig 6B). No recombination between the two major clades obtained from the mitochondrial genomes was identified, Phi test (p = 0.09032). However, the Phi test for recombination using the mitochondrial SNP data indicated that recombination was present within each of the two major VGIII serotype groups, serotype B, 498 SNPs, with 320 SNPs being parsimony informative, Phi test (p = 0.0032), and serotype C, 333 SNPs, with 258 being parsimony informative, Phi test (p = 0.000000143), indicating possible sexual recombination events.

### Serotype B prevails amongst the highly virulent VGIII isolates, causing pulmonary cryptococcosis

**Fig 8:**
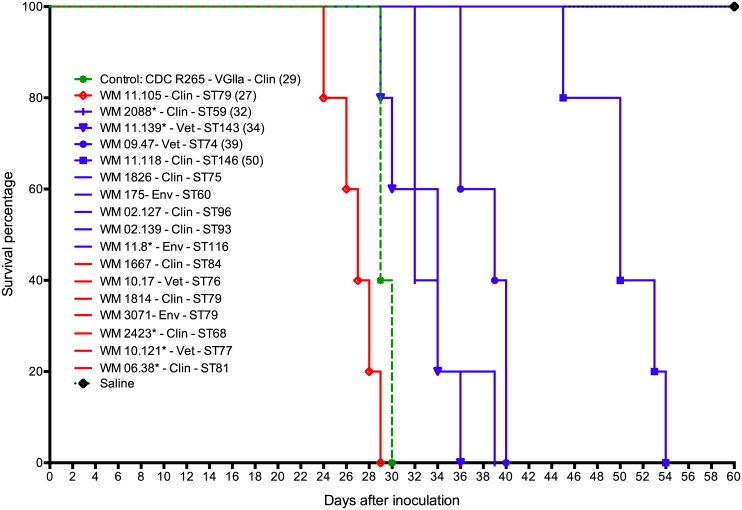
Survival curves of BALB/c mice inoculated with isolates of *Cryptococcus gattii* molecular type VGIII and the highly virulent VGIIa strain CDCR265 [69]. Blue lines indicates serotype B and red lines, serotype C. Asterisk (*) indicates mating type **a** isolates, all other isolates are mating type α. Clin: clinical and Vet: veterinary. The median survival time (in days) is indicated in brackets.

Seventeen isolates, widely representative of the identified MLST genotypes, were studied in a mouse model of infection. Five were highly virulent and caused 100% mortality, while 12 did not kill any mice within 60 days of inoculation (Table 2, Fig 8). Of the virulent isolates, WM 11.105 (C/α, ST79, a clinical isolate from Colombia) was the most virulent, even more lethal than CDC R265 (the highly virulent VGIIa reference strain from the Vancouver Island outbreak, which was used as a control [69]) (*p* = 0.0112), followed by WM 2088 (B/**a**, ST59, a clinical isolate from Colombia), WM 11.139 (B/**a**, ST143, a veterinary isolate from the USA), WM 09.47 (B/α, ST74, a veterinary isolate from the USA) and WM 11.118 (B/α, a clinical isolate from Colombia), which was the least virulent (*p* = 0.0025). Pairwise comparison among the other virulent isolates showed no significant differences (*p* >0.05). Four of the five virulent isolates were serotype B while only one was serotype C. Of the four serotype B isolates, two human clinical isolates (WM 11.139 and WM 2088) were mating type **a** and two veterinary isolates (WM 09.47 and WM 11.139) were mating type α. No environmental isolates were virulent in this mouse model.

**Table 2:** List of *Cryptococcus gattii* molecular type VGIII isolates tested in the murine model of infection (n = 17).

WM number	Country	Source	Serotype/ mating type	Median survival time (days)
WM 11.105	Colombia	Clinical	C/α	27*
WM 2088	Colombia	Clinical	B/**a**	32*
WM 11.139	USA	Veterinary	B/**a**	34*
WM 09.47	USA	Veterinary	B/α	39*
WM 11.118	Colombia	Clinical	B/α	50*
WM 1826	Mexico	Clinical	B/α	ND*
WM 175	USA	Environmental	B/α	ND
WM 02.127	Guatemala	Clinical	B/α	ND*
WM 02.139	USA	Clinical	B/α	ND*
WM 11.8	Colombia	Environmental	B/**a**	ND*
WM 1667	USA	Clinical	C/α	ND
WM 10.17	USA	Veterinary	C/α	ND*
WM 1814	Mexico	Clinical	C/α	ND
WM 3071	Colombia	Environmental	C/α	ND
WM 2423	New Zealand	Clinical	C/**a**	ND*
WM 10.121	USA	Veterinary	C/**a**	ND
WM 06.38	USA	Clinical	C/**a**	ND

ND: Not determined. These isolates did not kill any mouse at the end of the experiment (60 days). The reference strains of the highly virulent subtype VGIIa (CDCR265) and low virulent subtype VGIIb (CDCR272) were used as controls for infection *in vivo* [69]. The median survival of mice inoculated with CDCR265 was 29 days, where no mice infected with CDCR272 died within the 60-day observation period.

*Fungal cells were culture from cardiac blood collected at necropsy. This raises the possibility that fungal cells seen by histology in brain tissue might have been located within cerebral blood vessels rather than being present in the meninges or brain parenchyma.

**Fig 9:**
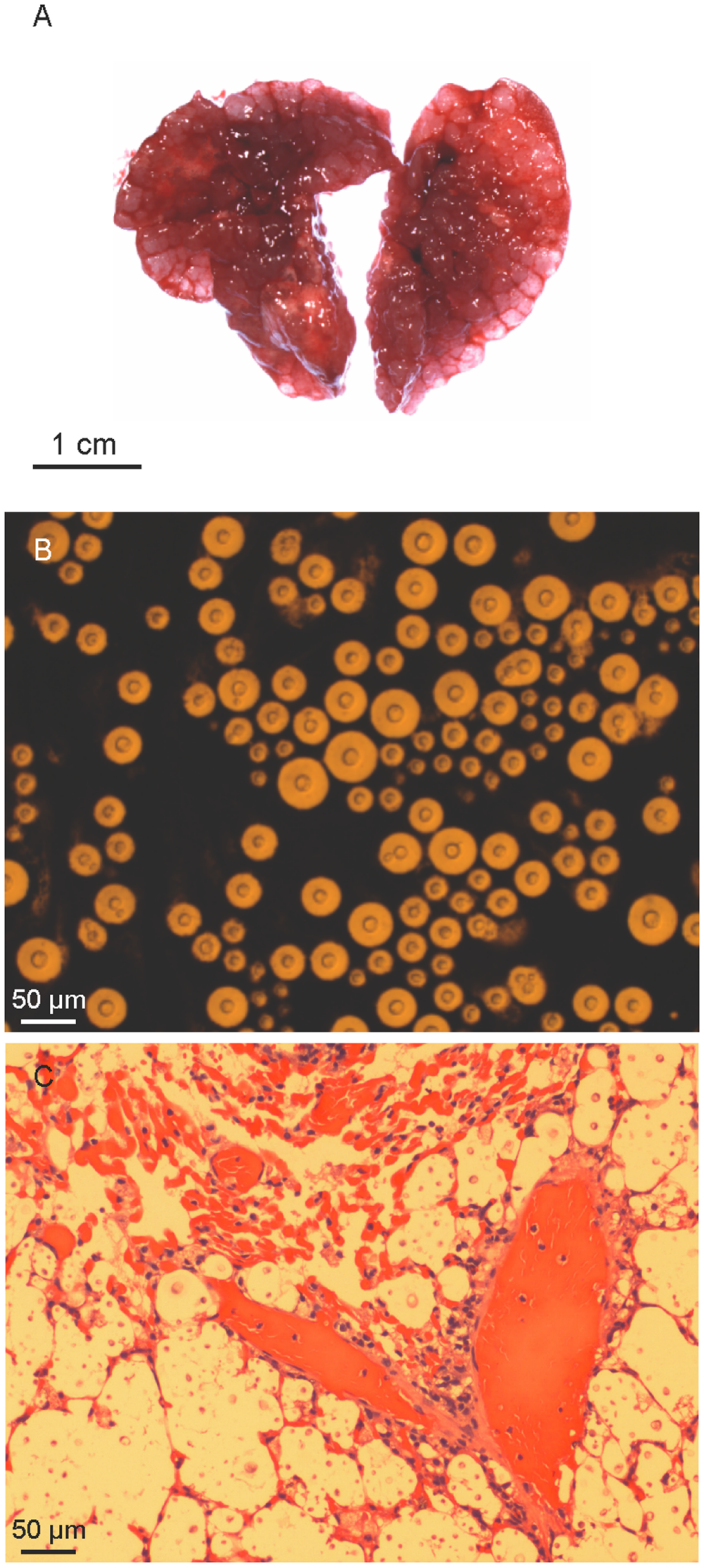
Pathological findings from BALB/c mice inoculated with a high virulent isolate of *Cryptococcus gattii* molecular type VGIII (WM 11.105). **A.** Lungs with multiple gelatinous granulomata comprised mostly of masses of cryptococcal cells (cryptococcomas). **B.** India ink stained lung suspension examined microscopically displaying abundant cryptococcal cells. **C.** Histopathological section of infected lungs stained with haematoxylin and eosin showing alveoli containing large numbers of yeast cells and little or no inflammatory response.

**Fig 10:**
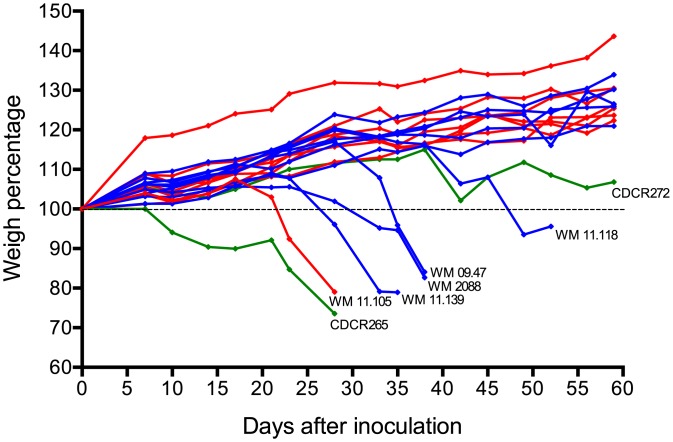
Change in body weight (expressed as a percentage) of BALB/c mice inoculated with *Cryptococcus gattii* molecular types VGII and VGIII. Blue lines indicates VGIII serotype B, red lines, VGIII serotype C and green lines, VGII isolates. Virulent VGIII isolates and isolates of subtypes VGIIa (CDCR265) and VGIIb (CDCR272) are labelled [69]. Code numbers of isolates that did not kill any mice at the end of the experiment are not included.

Macroscopic examination after necropsy revealed multiple granulomata in the lungs of the mice infected with virulent cryptococcal isolates. In contrast, few or no granulomata were observed in lung tissue from mice that survived for at least 60 days post inoculation. Direct microscopy of the lung tissue suspensions stained with Indian ink revealed numerous cryptococci in the lung samples of all infected mice. Lung tissue burdens of cryptococci (number of yeasts per gram) did not differ significantly among the virulent isolates. Brains excised from these mice were macroscopically normal and brain suspensions were culture negative. Direct microscopy of India ink preparations of the brain suspensions revealed not more than four cryptococcal cells. It is possible these represent yeasts originating from cerebral or meningeal blood vessels. Histological examination of lung tissue from mice infected with the five virulent isolates revealed widespread location of cryptococci within the alveoli, interstitial tissue and the airways. *C*. *gattii* was recovered from the cardiac blood from 11 out of the 17 isolates inoculated into mice, indicative of dissemination of cryptococcal cells from the lungs to circulation (Table 2). Fig 9 shows multiple granulomata and numerous cryptococci in the lungs of mice infected with the most virulent *C*. *gattii* VGIII isolate WM 11.105. Isolates with enhanced virulence caused significant weight loss during the course of infection (Fig 10).

### Serotype C isolates show less susceptibility to azoles than serotype B isolates

Antifungal susceptibilities of all VGIII isolates to amphotericin-B, 5-flucytosine, posaconazole, voriconazole, intraconazole, and fluconazole, were determined (S1 Table). Minimum inhibitory concentrations (MICs), MIC_50_, MIC_90_, geometric mean MICs and epidemiological cut-off values for all isolates are shown in Table 3. One veterinary isolate from the USA (WM 11.937) and two clinical isolates from Colombia (WM 11.105 and WM 11.112) had high fluconazole MICs; the first isolate (WM 11.937) with a MIC of 64 μg/ml and the last two (WM 11.105 and WM 11.112) with MICs of 128 μg/ml. The comparison of MIC distributions for the tested drugs according to the serotype, mating type and source of the isolates is shown in S4 Table. Overall, serotype C isolates had statistically significant higher modal MICs and geometric mean MICs for posaconazole, voriconazole, itraconazole and fluconazole than serotype B isolates, but lower geometric mean MICs for 5-fluorocytosine (*p* <0.05) (Table 4, S4 and S5 Tables). Statistically, environmental isolates were less susceptible to the tested antifungals, except for amphotericin-B, compared with clinical and veterinary isolates (Table 5 and S4 Table). There was no significant difference in antifungal susceptibility profiles between mating type α or **a** isolates (*p* > 0.05) (S4 Table). Susceptibility to amphotericin-B did not vary significantly with the source of the isolates (S4 Table). The epidemiological cut off values (ECVs) were the same as the MIC_90_ for the tested drugs, except for posaconazole, where the ECV was 0.25 μg/ml, compared with an MIC_90_ of 0.125 μg/ml (Table 3).

**Table 3:** Antifungal susceptibility results of the *Cryptococcus gattii* molecular type VGIII isolates (n = 122) determined by microdilution (Sensititre YeastOne).

	MIC (mg/ml)^a^					
	AMB	FC	PCZ	VCZ	ITZ	FCZ
Range	≤0.125–2	0.5–8	0.015–0.25	≤0.008–1	≤0.015–2	1–128
MIC_50_	0.25	2	0.06	0.03	0.03	4
MIC_90_	0.5	4	0.125	0.125	0.125	16
Geometric mean^b^	0.2865	1.879	0.06923	0.04249	0.04573	5.224
ECV^c^	0.5 (98.4)	4 (95.9)	0.25 (95.1)	0.125 (95.9)	0.125 (98.4)	16 (95.9)

^a^AMB: Amphotericin-B; FC: 5-Fluorocytosine; PCZ: Posaconazole, VCZ: Voriconazole; ITZ: Itraconazole; FCZ: Fluconazole

^b^Geometric mean MIC

^c^Epidemiologic cutoff value (% wild type distribution)

**Table 4:** Antifungal susceptibility of the *Cryptococcus gattii* molecular type VGIII isolates by serotype.

	MIC (μg/ml)			
Serotype	Range	MIC_50_	MIC_90_	Geometric mean
Amphotericin B (*p* = 0.1153)				
Serotype B	≤0.125–1	0.25	0.5	0.2980
Serotype C	≤0.125–2	0.25	0.5	0.2691
5-Flucytosine (*p* = 0.0397)*				
Serotype B	0.5–8	2	4	2.095
Serotype C	0.5–4	2	4	1.580
Posaconazole (*p* < 0.0001)****				
Serotype B	0.015–0.25	0.06	0.125	0.05698
Serotype C	0.03–0.25	0.125	0.125	0.09445
Voriconazole (*p* < 0.0001)****				
Serotype B	≤0.008–1	0.03	0.06	0.03304
Serotype C	≤0.008–0.5	0.06	0.125	0.06346
Itraconazole (*p* = 0.0031)**				
Serotype B	≤0.015–0.25	0.03	0.125	0.03830
Serotype C	≤0.015–2	0.06	0.125	0.06068
Fluconazole (*p* < 0.0001)****				
Serotype B	1–128	4	8	3.927
Serotype C	1–128	8	32	8.239

Statistical significance * *p* ≤ 0.05, ** *p* ≤ 0.01, *** *p* ≤ 0.001 and **** *p* ≤ 0.0001

**Table 5:** Geometric mean MICs of the *Cryptococcus gattii* molecular type VGIII isolates according to source.

	Geometric mean MIC (μg/ml)^a^					
Source (n)	AMB	FC	PCZ	VCZ	ITZ	FCZ
Environmental (28)	0.2563	2.208	0.09520	0.5666	0.06732	7.246
Clinical (56)	0.2726	1.927	0.06987	0.04421	0.04530	5.384
Veterinary (38)	0.3347	1.607	0.05401	0.03241	0.03486	3.928

^a^AMB: Amphotericin-B; FC: 5-Flucytosine; PCZ: Posaconazole, VCZ: Voriconazole; ITZ: Itraconazole; FCZ: Fluconazole

## Discussion

Taking into account the rising importance of VGIII as cause of clinical and veterinary infections [17, 20–24, 27–29], their genotypic and phenotypic epidemiology has been under-investigated, compared with strains of molecular type VGII. Two MLST studies performed on a VGIII population from HIV positive patients from Southern California, identified two major molecular groups, VGIIIa and VGIIIb, that differed in virulence and fertility and a minor VGIIIc cluster represented by only one isolate [20,32]. Shortly after the first study was published, VGIII was found to predominate among the molecular types recovered from both human and veterinary samples outside the Pacific Northwest in the USA [17], and amongst cats in California [28]. It was also identified as the second most common molecular type amongst human cases in Colombia [24]. Based on these limited reports, we conducted a VGIII population analysis of clinical, veterinary and environmental isolates from a broader geographic range, taking into account the previously reported endemic areas and sporadic cases [25]. Isolates were sampled from the USA, Colombia and Mexico, and single cases from Australia, Guatemala, New Zealand, Paraguay and Venezuela, to give a more comprehensive perspective of the epidemiology of the VGIII molecular type and to investigate possible correlations between genotype and virulence and antifungal susceptibility phenotypes.

Although *C*. *gattii* VGIII has been recovered infrequently from human cases in Argentina, Guatemala [8], Cuba [37], Western Europe [72] and Korea [73, 74], and from the environment in Argentina [31] and India [30], this molecular type remains an important cause of neglected cryptococcosis cases in Brazil [34, 38], Colombia [23,24] and Mexico [21,35]. In addition, the previously described endemic region of *C*. *gattii* VGIII is expanding beyond the borders of the US state of California, with an increase of both clinical and veterinary cases [17,28]. The high incidence of cryptococcosis caused by *C*. *gattii* VGIII beyond the tropical and subtropical areas, where it is considered to be endemic, and the emergence of this pathogen in more temperate regions traditionally considered of low risk for the acquisition of this infection are major clinical concerns. *C*. *gattii*, including VGIII is mostly isolated from patients without recognized immunologic defects and may be associated with worse clinical outcomes, complications such as permanent neurologic sequelae and the requirement for prolonged periods of antifungal treatment [37,39–41]. Failure to consider the diagnosis or delays in doing so in immunocompetent patients, result in failing to initiate the most appropriate therapy, and consequently increase morbidity and mortality. This is illustrated by recent cases of disseminated cryptococcosis caused by *C*. *gattii* VGIII, including two fatal cases reported from Cuba and the USA [37,39].

As also reported in the aforementioned studies, the herein studied VGIII population showed a high level of genetic diversity, with no geographic restriction of genotypes. This is at variance with observations made with VGII subtypes [14–17]. Not only were the VGIII isolates from the endemic areas of Colombia, Mexico and the USA closely related, but they also shared genotypes with most of the sporadic cases from around the world, namely ST68 (found in New Zealand and the USA), and ST65 (found in Venezuela and Colombia) (Figs 1 and 4). Although only a single isolate from Guatemala was identified as ST96, this genotype clustered very closely with isolates from Mexico (Fig 1). Similarly, a single isolate of ST144 found in Australia, clustered very closely with isolates from the USA (Fig 1). Interestingly, there was an association between clinical and veterinary genotypes and those identified from environmental samples, which present the natural reservoirs for *C*. *gattii* (Fig 1).

Determination of the serotype of the isolates clearly revealed that the major MLST, WGST and mitochondrial genome clusters within VGIII correspond to either serotype B or serotype C, corresponding most likely to the subgroups VGIIIa and VGIIIb, respectively, which were designated previously, based exclusively on MLST genotypic clustering [20,32]. This discrimination between the serotypes of *C*. *gattii* VGIII has been demonstrated previously using Fourier transform infrared spectroscopy, which, in contrast to MLST, WGS and mitochondrial genome sequencing, characterizes phenotype instead of genotype [72]. Phylogenetic and coalescence analyses also revealed two more distant, but basal groups in the VGIII population, which interestingly, have been erroneously designated as VGIV following *URA5*-RLFP analysis, due to a SNP in the restriction site of *Sau*96I [12]. In addition, these two minor clusters did not share any of the MLST alleles with the major groups. Notably, they were additionally separated according to serotype, in spite of being represented by only a few isolates each (Figs 1 and 2; S1 Table). Importantly, one of these minor/basal groups, which includes the serotype C isolates WM 1802 and WM 1804 from Mexico, is closely related to the previously described AFLP10 type, which differs in one of the seven MLST loci, the *SOD1* locus having allele type (AT) AT51 in strain IHEM14941S compared to AT39 in the herein studied strains WM 1802 and WM 1804 [11,32]. This strain has recently been proposed as a distinct species among *C*. *gattii* (*C*. *decagattii*) [13]. The second minor group described in this study, which includes the serotype B isolates WM 2004, WM 2041 and WM 2042 (all ST64) from Colombia, and WM 11.32 (ST114) from the USA, could similarly represent another new cryptic species, considering the species concept proposed by Hagen *et al*. [13]. However, DNA barcoding gap analysis, accounting for all four identified subgroups within the VGIII isolates combined, does not reveal a DNA barcoding gap (S3A Fig). If the newly described minor serotype B subgroup (WM 2004, WM 2041, WM 2042 and WM 11.32) is removed from this analysis, a DNA barcoding gap emerges between the major two serotype groups, B and C, and the minor serotype C subgroup (WM 1802 and WM 1804, similar to AFLP10 [13]) (S3B Fig). As such, the separation of the AFLP10 isolates from the VGIII isolates may be mistaken because of the small sample size. Depending on the number of isolates that are included in the phylogenetic analysis, the species borders can become blurred, indicating ongoing speciation events.

Based solely on the MLST analysis serotype C isolates were slightly more diverse than serotype B isolates and there was a low gene flow between isolates of different serotypes, as reflected by population differentiation analysis (Figs 1, 2 and 3). Tests of linkage disequilibrium showed additionally that serotype C isolates recombine more readily than serotype B isolates. These conclusions were also supported by the phylogenetic network and *fineStructure* analyses of the whole genomes and the mitochondrial genomes, showed almost no recombination between the two serotypes but recombination within each of the serotypes, with more sharing of genomic content amongst serotype C isolates (Figs 6 and 7). Identification of the two opposite mating types amongst both serotype B and serotype C isolates provides further evidence for sexual recombination within these VGIII sub-populations, which may contribute to their genetic diversity. The occurrence of well-supported sub-populations, which are separated geographically and in time, suggests that recombination and genetic exchange events are not occurring between the two major serotype specific groups of the molecular type VGIII and that this population is going through a process of expansion, divergence and perhaps speciation.

MLST, maximum parsimony-based WGST, and coalescence analyses demonstrated that the two major VGIII sub-populations, serotypes B and C, which share minimal genetic diversity, likely originated from very distant ancestors in the VGIII endemic regions of Colombia, Mexico or the USA (Figs 1A, 1B, 4 and 5). The two distinct atypical populations link the two major VGIII populations (serotype B and C) specifically to the *C*. *gattii* lineages VGI and VGIV, confirming findings from comparative WGS studies, which also showed a closer link between VGI, VGIII and VGIV [75] (Fig 5). *fineStructure* analysis confirmed their ancestral role by indicating shared gene content between VGI and/or VGIV (S2 Fig). This reflects also findings by *Farrer at al*., that structural genome rearrangements are almost exclusive to the VGI, VGIII and VGIV lineages [75]. Given that atypical isolates may be under-sampled or misidentified as molecular type VGIV using traditional molecular methods (i.e. *URA5*-RFLP), further studies are required to more accurately infer the ancestors of the VGIII population.

Inclusion of the highly virulent reference strain of the VGIIa subtype (CDCR265) [69] in the mouse model of infection permitted the recognition of VGIII isolates with enhanced or comparable virulence to the VGII Vancouver Island outbreak isolates and very similar diseases patterns (Figs 8, 9 and 10; Table 2). Importantly, the mortality from infection with serotype B isolates was higher than that caused by serotype C isolates. Nevertheless, most of the isolates formed granulomata and direct microscopic examination revealed yeast cells in all lung sections similar to an isolate with increased virulence (WM 11.105) (Fig 9). These findings indicate that as reported in infections mainly caused by VGIIa strains in British Columbia and the Pacific Northwest [36,76], pulmonary cryptococcosis is the predominant clinical manifestation of *C*. *gattii* VGIII serotype B infections. Among the highly virulent serotype B isolates identified in this study, the veterinary isolate WM 09.47 shared the same genotype (ST74) with the strain responsible for a fatal case of cryptococcosis reported in New Mexico in 2010 (WM 11.935, B7495) [39] and with a clinical isolate (WM 1819) from Mexico recovered in 1990. This finding suggests that the identification of certain genotypes may be indicative of increased virulence. It is conceivable that these virulent genotypes are circulating but are undocumented. Paradoxically, there were no specific whole or mitochondrial genome differences between low- and high-virulence isolates in the two major groups. This is similar to the findings by Ma *et al*. in 2009, which showed also no correlation between the major genes coding for known virulence factors and the actual virulence in the VGII Vancouver Island Outbreak strains [70], but identified changes in the mitochondrial morphology and mitochondrial gene expression as major factors of increased intracellular proliferation, corresponding to increased virulence. However, specific comparative mitochondrial genome analysis between high and low virulent VGIII strains conducted herein did not, like the WGS analysis, reveal any specific changes. Gene rearrangement analysis (i.e., progressive Mauve) showed variation among the mitochondrial genomes of the strains included in the virulent study, not specific changes associated with either high or low virulent strains were found (S4 Fig). The variation found within the mitochondrial genomes is in agreement with the observation made previously, attributing the fact that mitochondria are more recombinogenic than their nuclear counterparts, to the ability to change the mitochondrial phenotype [75]. As no specific whole genome or mitochondrial genome differences between high and low virulent have been found herein, the differences in virulence may therefore be related to phenotypic characteristics generated by differences in gene expression, for example, different rates of multiplication at 37°C, the ability to disseminate from the lung to the brain and other sites via the blood and to overcome the host immune response.

Recovery of yeast cells from cardiac blood (heart blood collected at time of euthanasia) suggests that *C*. *gattii* VGIII can disseminate to the CNS, but that experimentally-inoculated mice die of cryptococcal pneumonia before establishment of meningoencephalitis, as previously found [77]. We suggest that the small number of yeasts detected by direct examination of brain tissue from mice infected with VGIII isolates represented yeasts within cerebral blood vessels, as there was no clinical or histopathological evidence for infection of the central nervous system.

To date, many *in vitro* susceptibility studies have been performed on *C*. *neoformans* and *C*. *gattii*, to elucidate differences between species, serotypes and molecular types. Differences between serotypes have been mostly reported within *C*. *neoformans*, where there is a clear correspondence between serotype and molecular type. This close correspondence has not been reported within *C*. *gattii*, mainly because the serotype in this species is rarely identified and documented [11,28,42,43,78–81]. Because differences in antifungal susceptibility can influence therapeutic choices in the clinical setting, the findings of this study are of interest. Serotype C isolates were significantly less susceptible to azoles, especially fluconazole, than serotype B isolates (Table 4). In addition, irrespective of serotype, environmental isolates were slightly less susceptible to azoles and 5-fluorocytosine than clinical and veterinary isolates, indicating that their use in establishing ECVs may be misleading (Table 5). However, the association between the source of the isolates (clinical, veterinary and environmental) and antifungal susceptibility profiles remains unclear, as different findings have been reported elsewhere. In a previous Brazilian study, for instance, clinical isolates of *C*. *neoformans* were reported to be less susceptible to antifungal drugs than environmental isolates [80], and in general, veterinary isolates of *C*. *gattii* collected worldwide were found to be less susceptible to antifungal drugs than clinical and environmental isolates [11].

Overall, *C*. *gattii* VGIII strains have been more susceptible to amphotericin B and 5-flucytosine than other *C*. *gattii* molecular types and *C*. *neoformans* [42,78,81]. In the present study ECVs of both these antifungal agents were higher than reported previously (0.5 μg/ml for amphotericin B and 4 μg/ml for 5-flucytosine). *C*. *gattii* has shown variable susceptibility to fluconazole and other azoles [28,43,79]. However, this study is the first to document high values of GM MICs and ECVs amongst *C*. *gattii* VGIII isolates, specifically for fluconazole and itraconazole, which are currently recommended as alternative induction therapy for pulmonary cryptococcosis [82]. All of these findings emphasize that recognition of serotype and molecular type in *C*. *gattii* isolates can identify isolates with acquired resistance mechanisms, based on the reported ECVs for each drug and may be relevant to the choice of the treatment regimen for a specific cryptococcal infection. Clinical studies correlating these parameters with responses to therapy and patient outcomes are required.

In conclusion, the herein reported study of clinical, veterinary and environmental isolates from the main endemic areas in the world revealed a high genetic diversity within the *C*. *gattii* molecular type VGIII population. Two well-supported and divergent lineages were identified, corresponding to serotypes B and C. In addition distant ancestors within the molecular type that are represented by isolates from VGIII endemic areas were revealed in either Mexico or Colombia/USA, linking the two major VGIII populations to the other major molecular types within *C*. *gattii*, specifically to VGI and VGIV. The predominant clinical manifestation of *C*. *gattii* VGIII infections was pulmonary disease rather than meningitis or encephalitis. No specific associations between the WGS or mitochondrial genome and virulence have been observed. Antifungal susceptibility profiles differed according to serotype. The results of this study reinforce the notion that global cooperation is necessary to more accurately determine the prevalence of *C*. *gattii* infection and redefine endemic regions. Additionally, surveillance of antifungal susceptibility patterns and correlation with clinical outcomes is needed to optimize therapeutic guidelines and hence clinical outcomes.

## Supporting Information

S1 Table List of studied *Cryptococcus gattii* molecular type VGIII isolates. General strain information, MLST results and antifungal susceptibility results, are presented. Isolates are organized according to sequence type (ST).
(DOC)

S2 Table Deposited allele sequences. GenBank accession numbers for the allele types of *Cryptococcus gattii* molecular type VGIII isolates.
(DOC)

S3 Table Genotypic diversity. Diversity of the sequence types (STs) of *Cryptococcus gattii* molecular type VGIII isolates (n = 122).
(DOC)

S4 Table Antifungal susceptibility differences. Comparison of MIC values for significant differences between *Cryptococcus gattii* molecular type VGIII isolates (n = 122).
(DOC)

S5 Table MIC values per serotype. Distribution of MIC values of *Cryptococcus gattii* molecular type VGIII isolates by serotype.
(DOC)

S1 Fig Dendrogram of the partial sequences of the *CAP59* gene. The tree is based on the analysis of a fragment of 360 bp of the capsular gene extracted from the WGS data using the program MEGA 6.0 [48] and indicating the serotype B (blue) and serotype C (red) isolates.
(EPS)

S2 Fig *fineStructure* analysis of all sub-populations of *Cryptococcus gattii* VGIII in context with the other *C*. *gattii* molecular types: VGI, VGII and VGIV. To identify the population structure and genome exchange events between the VGIII groups and the remaining *C*. *gattii* molecular types, *fineStructure* analysis was performed using a SNP matrix (i). Whole-genome SNP data were reduced to a pairwise similarity matrix. The x-axis represents the strain as a “donor” and the y-axis represents the strain as a “recipient” of genomic regions. The scale bar represents the number of shared genome regions with blue being the greatest amount of sharing and yellow being the least. Blue and red boxes represent the main VGIII serotype B and serotype C isolates, respectively.
(TIF)

S3 Fig Barcoding gap analysis. **(A)** Identified subgroups within the VGIII isolates combined do not reveal a DNA barcoding gap. **(B)** If the newly described minor serotype B subgroup (WM 2004, WM 2041, WM 2042 and WM 11.32) is removed from this analysis, a DNA barcoding gap emerges between the major two serotype groups, B and C, and the minor serotype C subgroup (WM 1802 and WM 1804, similar to AFLP10).
(EPS)

S4 Fig progressiveMauve analysis of the mitochondrial genomes. High virulent: WM 09.47, WM 11.118, WM 11.139, WM 2088, and low virulent: WM 11.8, WM 02.1`39, WM 02.127, WM 18260 *C*. *gattii* VGIII strains.
(JPEG)
