# Supplementary material for: MLST and Whole-Genome-Based Population Analysis of Cryptococcus gattii VGIII Links Clinical, Veterinary and Environmental Strains, and Reveals Divergent Serotype Specific Sub-populations and Distant Ancestors
Source: PLoS Negl Trop Dis. 2016 Aug 5;10(8):e0004861. doi: 10.1371/journal.pntd.0004861 (PMC4975453; doi:10.1371/journal.pntd.0004861)
Supplement: S1 Table — General strain information, MLST results and antifungal susceptibility results, are presented. Isolates are organized according to sequence type (ST). (DOC) [file pntd.0004861.s001.doc]

**S1 Table**. **List of studied *Cryptococcus gattii* molecular type VGIII isolates.** General strain information, MLST results and antifungal susceptibility results, are presented. Isolates are organized according to sequence type (ST).

|  |  |  |  |  |  | **Alleles and sequence types** | | | | | | | | **MIC (µg/ml)^d^** | | | | | |  |
| --- | --- | --- | --- | --- | --- | --- | --- | --- | --- | --- | --- | --- | --- | --- | --- | --- | --- | --- | --- | --- |
| **WM number** | **Other collection number** | **Country^a^** | **Source^b^** | **Year** | **Serotype^c^/**  **Mating type** | ***CAP59*** | ***GPD1*** | **IGS1** | ***LAC1*** | ***PLB1*** | ***SOD1*** | ***URA5*** | **ST** | **AMB** | **FC** | **PCZ** | **VCZ** | **ITZ** | **FCZ** | **Ref^e^** |
| WM 09.43 | JS27 | USA | Vet | 2006 | B/α | 18 | 3 | 1 | 33 | 6 | 40 | 19 | 72 | 0.5 | 4 | 0.06 | 0.03 | 0.06 | 4 | [1] |
| WM 11.137 | JS108 | USA | Vet | 2011 | B/α | 18 | 3 | 1 | 3 | 6 | 40 | 19 | 75 | 0.5 | 4 | 0.06 | 0.03 | ≤0.015 | 2 | JS |
| WM 11.129 | JS100 | USA | Vet | 2011 | B/α | 18 | 3 | 1 | 3 | 6 | 40 | 19 | 75 | 0.25 | 1 | 0.06 | 0.03 | ≤0.015 | 4 | JS |
| WM 11.939 | B8516 | USA | Vet | 2009 | B/α | 18 | 3 | 1 | 3 | 6 | 40 | 19 | 75 | 0.25 | 2 | 0.03 | 0.03 | ≤0.015 | 2 | [2] |
| WM 10.176 | JS54 | USA | Vet | 2006 | B/α | 18 | 3 | 1 | 3 | 6 | 40 | 19 | 75 | 0.5 | 8 | 0.25 | 0.125 | 0.25 | 8 | [1] |
| WM 1826 | LA 414; INDRE 5631 | MEX | Clin | 1999 | B/α | 18 | 3 | 1 | 3 | 6 | 40 | 19 | 75 | 0.5 | 4 | 0.06 | 0.03 | 0.06 | 4 | LC |
| WM 10.165 | JS5 | USA | Vet | 2005 | B/α | 18 | 3 | 1 | 3 | 6 | 40 | 19 | 75 | 0.25 | 1 | 0.03 | 0.03 | 0.03 | 4 | JS |
| WM 11.936 | B8212 | USA | Clin | 2009 | B/α | 18 | 3 | 1 | 3 | 6 | 40 | 19 | 75 | ≤0.12 | 2 | 0.125 | 0.06 | 0.125 | 8 | [3] |
| WM 11.126 | JS97 | USA | Vet | 2011 | B/α | 18 | 3 | 1 | 3 | 6 | 40 | 19 | 75 | 0.5 | 2 | 0.03 | 0.03 | 0.03 | 4 | JS |
| WM 11.130 | JS101 | USA | Vet | 2011 | B/α | 18 | 3 | 1 | 3 | 6 | 40 | 19 | 75 | 0.25 | 1 | 0.03 | 0.015 | ≤0.015 | 2 | JS |
| WM 11.133 | JS104 | USA | Vet | 2011 | B/α | 18 | 3 | 1 | 3 | 6 | 40 | 19 | 75 | 0.25 | 1 | 0.06 | 0.015 | 0.03 | 4 | JS |
| WM 11.949 | B9322 | USA | Clin | 2011 | B/α | 18 | 3 | 1 | 3 | 6 | 40 | 19 | 75 | 0.25 | 8 | 0.06 | 0.06 | 0.06 | 8 | [3] |
| WM 11.947 | B9243 | USA | Clin | 2011 | B/α | 18 | 3 | 1 | 3 | 6 | 40 | 19 | 75 | 0.5 | 2 | 0.06 | 0.03 | 0.03 | 4 | [3] |
| WM 11.953 | B9372 | USA | Vet | 2011 | B/α | 18 | 3 | 1 | 3 | 6 | 40 | 19 | 75 | 0.25 | 2 | 0.06 | 0.06 | 0.06 | 8 | [3] |
| WM 09.48 | 08-6825 | USA | Vet | 2008 | B/α | 18 | 3 | 1 | 3 | 6 | 40 | 19 | 75 | 0.25 | 2 | 0.06 | 0.06 | 0.06 | 4 | [1] |
| WM 10.161 | JS1 | USA | Vet | 2005 | B/α | 18 | 3 | 1 | 3 | 6 | 40 | 19 | 75 | 0.5 | 2 | 0.03 | 0.015 | 0.03 | 4 | JS |
| WM 10.163 | JS3 | USA | Vet | 2005 | B/α | 18 | 3 | 1 | 3 | 6 | 40 | 19 | 75 | 0.5 | 4 | 0.06 | 0.03 | ≤0.015 | 2 | JS |
| WM 11.934 | B7415 | USA | Vet | 2009 | B/α | 18 | 3 | 1 | 3 | 6 | 40 | 19 | 75 | 0.25 | 1 | 0.06 | 0.03 | 0.03 | 4 | [2] |
| WM 11.940 | B8964 | USA | Clin | 2010 | B/α | 18 | 3 | 1 | 3 | 6 | 40 | 19 | 75 | ≤0.12 | 4 | 0.125 | 0.06 | 0.125 | 8 | [2] |
| WM 11.944 | B9148 | USA | Clin | 2011 | B/α | 18 | 3 | 1 | 3 | 6 | 40 | 19 | 75 | ≤0.12 | 1 | 0.03 | 0.015 | 0.03 | 2 | [3] |
| WM 11.37 | JS86 | USA | Vet | 2011 | B/α | 18 | 3 | 1 | 3 | 6 | 40 | 19 | 75 | 0.25 | 1 | 0.06 | 0.03 | ≤0.015 | 2 | JS |
| WM 11.132 | JS103 | USA | Vet | 2011 | B/α | 18 | 3 | 1 | 3 | 6 | 40 | 19 | 75 | 0.5 | 4 | 0.03 | 0.015 | ≤0.015 | 2 | JS |
| WM 10.181 | JS61 | USA | Vet | 2006 | B/α | 18 | 3 | 1 | 3 | 35 | 40 | 19 | 115 | 0.5 | 2 | 0.06 | 0.015 | ≤0.015 | 4 | JS |
| WM 1661 | UCLA373B | USA | Clin | <1965 | B/α | 18 | 3 | 1 | 3 | 6 | 40 | 21 | 87 | 0.5 | 2 | 0.06 | 0.03 | 0.03 | 2 | [1] |
| WM 11.139 | JS110 | USA | Vet | 2011 | B/**a** | 18 | 3 | 1 | 3 | 6 | 40 | 23 | 143 | 0.25 | 1 | 0.015 | 0.015 | ≤0.015 | 2 | JS |
| WM 11.35 | JS84 | USA | Vet | 2011 | B/**a** | 18 | 3 | 1 | 3 | 6 | 40 | 23 | 143 | 0.25 | 1 | 0.03 | 0.03 | 0.03 | 2 | JS |
| WM 11.30 | JS79 | USA | Vet | 2010 | B/**a** | 18 | 3 | 1 | 3 | 6 | 40 | 23 | 143 | 0.5 | 2 | 0.06 | 0.03 | 0.03 | 4 | JS |
| WM 2358 | ATCC 34878; NIH 179 | USA | Clin | 1981 | B/α | 18 | 3 | 1 | 15 | 6 | 40 | 23 | 89 | 0.25 | 2 | 0.06 | 0.03 | 0.03 | 4 | [4] |
| WM 11.951 | B9354 | USA | Clin | 2011 | B/α | 18 | 3 | 1 | 15 | 6 | 40 | 19 | 139 | 0.25 | 2 | 0.06 | 0.03 | 0.06 | 4 | [3] |
| WM 11.942 | B9143 | USA | Clin | 2011 | B/α | 18 | 3 | 1 | 15 | 6 | 40 | 19 | 139 | 0.25 | 4 | 0.015 | 0.015 | ≤0.015 | 2 | [3] |
| WM 728 | TP0686; 22686 | USA | Env | 1992 | B/α | 18 | 18 | 14 | 3 | 6 | 28 | 19 | 60 | 0.25 | 2 | 0.06 | 0.03 | 0.03 | 4 | [4] |
| WM 726 | TP0696; 22696 | USA | Env | 1993 | B/α | 18 | 18 | 14 | 3 | 6 | 28 | 19 | 60 | 0.25 | 2 | 0.06 | 0.03 | 0.03 | 4 | [4] |
| WM 175 | CBS 10081 | USA | Env | 1992 | B/α | 18 | 18 | 14 | 3 | 6 | 28 | 19 | 60 | 0.25 | 1 | 0.06 | 0.03 | 0.03 | 4 | [4] |
| WM 11.8 | H0058-I-2728 | COL | Env | 2006 | B/**a** | 18 | 3 | 1 | 3 | 6 | 38 | 19 | 116 | 0.25 | 4 | 0.03 | ≤0.008 | ≤0.015 | 1 | PE |
| WM 11.9 | H0058-I-2729 | COL | Env | 2006 | B/**a** | 18 | 3 | 1 | 3 | 6 | 38 | 19 | 116 | 0.25 | 4 | 0.06 | 0.03 | 0.03 | 4 | PE |
| WM 11.10 | H0058-I-2730 | COL | Env | 2006 | B/**a** | 18 | 3 | 1 | 3 | 6 | 38 | 19 | 116 | 0.25 | 4 | 0.125 | 0.06 | 0.125 | 8 | PE |
| WM 11.11 | H0058-I-2731-1 | COL | Env | 2006 | B/**a** | 18 | 3 | 1 | 3 | 6 | 38 | 19 | 116 | 0.25 | 8 | 0.06 | 0.03 | 0.06 | 4 | PE |
| WM 11.12 | H0058-I-2748 | COL | Env | 2006 | B/**a** | 18 | 3 | 1 | 3 | 6 | 38 | 19 | 116 | ≤0.12 | 2 | 0.06 | 0.03 | 0.06 | 4 | PE |
| WM 11.13 | H0058-I-2749 | COL | Env | 2006 | B/**a** | 18 | 3 | 1 | 3 | 6 | 38 | 19 | 116 | 0.25 | 4 | 0.06 | 0.03 | 0.06 | 4 | PE |
| WM 11.14 | H0058-I-2750 | COL | Env | 2006 | B/**a** | 18 | 3 | 1 | 3 | 6 | 38 | 19 | 116 | 0.25 | 4 | 0.125 | 0.06 | 0.125 | 8 | PE |
| WM 2088 | H0058-I-1134; LA 622 | COL | Clin | 2000 | B/**a** | 18 | 3 | 1 | 3 | 17 | 38 | 19 | 59 | 0.25 | 2 | 0.06 | 0.03 | 0.03 | 4 | [5] |
| WM 11.104 | H0058-I-1708 | COL | Clin | 2003 | B/**a** | 18 | 3 | 1 | 3 | 17 | 38 | 19 | 59 | 0.5 | 4 | 0.125 | 0.06 | 0.125 | 8 | [5] |
| WM 1618 | LA 3; NUAM 122 | MEX | Clin | 1986 | B/α | 18 | 3 | 1 | 22 | 6 | 28 | 19 | 92 | 0.25 | 1 | 0.125 | 0.06 | 0.125 | 16 | LC |
| WM 1815 | LA 403; INDRE 5620 | MEX | Clin | 1986 | B/α | 18 | 3 | 1 | 22 | 6 | 28 | 19 | 92 | 0.5 | 2 | 0.125 | 0.125 | 0.125 | 16 | LC |
| WM 1620 | LA 5; NUAM 190 | MEX | Clin | 1995 | B/α | 18 | 3 | 23 | 20 | 17 | 28 | 29 | 97 | 0.25 | 1 | 0.03 | 0.015 | 0.03 | 2 | [4] |
| WM 1631 | LA 16; NUAM 221 | MEX | Clin | 1996 | B/α | 18 | 3 | 23 | 22 | 17 | 28 | 19 | 98 | 0.25 | 4 | 0.06 | 0.03 | ≤0.015 | 4 | LC |
| WM 1635 | LA 20; NUAM 227 | MEX | Clin | 1997 | B/α | 18 | 3 | 1 | 22 | 21 | 28 | 19 | 91 | 0.5 | 2 | 0.06 | 0.03 | ≤0.015 | 2 | LC |
| WM 1819 | LA 407; INDRE 5624 | MEX | Clin | 1990 | B/α | 18 | 3 | 1 | 32 | 20 | 40 | 23 | 74 | 0.25 | 0.5 | 0.06 | 0.03 | ≤0.015 | 4 | LC |
| WM 09.47 | 08-7686 | USA | Vet | 2008 | B/α | 18 | 3 | 1 | 32 | 20 | 40 | 23 | 74 | 0.25 | 0.5 | 0.015 | 0.015 | ≤0.015 | 1 | [1] |
| WM 10.178 | JS57 | USA | Vet | 2006 | B/α | 18 | 3 | 1 | 32 | 20 | 40 | 23 | 74 | 0.5 | 2 | 0.015 | 0.015 | ≤0.015 | 4 | [1] |
| WM 11.935 | B7495 | USA | Clin | 2009 | B/α | 18 | 3 | 1 | 32 | 20 | 40 | 23 | 74 | 0.25 | 1 | 0.06 | 0.03 | 0.03 | 8 | [2] |
| WM 11.33 | JS82 | USA | Vet | 2010 | B/α | 18 | 3 | 1 | 32 | 20 | 40 | 23 | 74 | 0.25 | 1 | 0.03 | 0.03 | 0.03 | 2 | [1] |
| WM 09.45 | 07-11763 | USA | Vet | 2007 | B/α | 18 | 3 | 1 | 34 | 17 | 48 | 19 | 73 | 0.25 | 1 | 0.03 | 0.015 | ≤0.015 | 2 | [1] |
| WM 11.124 | JS95 | USA | Vet | 2011 | B/α | 18 | 3 | 1 | 34 | 17 | 38 | 19 | 145 | 0.5 | 4 | 0.03 | 0.015 | 0.03 | 2 | [1] |
| WM 11.42 | JS93 | USA | Vet | 2011 | B/α | 18 | 3 | 1 | 34 | 17 | 38 | 19 | 145 | 0.5 | 8 | 0.06 | 0.03 | 0.03 | 4 | [1] |
| WM 1824 | LA 412; INDRE 5629 | MEX | Clin | 1997 | B/α | 18 | 3 | 1 | 20 | 17 | 28 | 29 | 90 | 1 | 4 | 0.03 | 0.015 | 0.03 | 2 | LC |
| WM 02.127 | LA 750 | GTM | Clin | 2000 | B/α | 18 | 3 | 5 | 20 | 17 | 28 | 29 | 96 | ≤0.12 | 2 | 0.03 | 0.03 | ≤0.015 | 2 | [4] |
| WM 1663 | UCLA385B | USA | Clin | <1965 | B/α | 18 | 9 | 5 | 14 | 6 | 28 | 21 | 94 | 0.25 | 4 | 0.25 | 0.06 | 0.125 | 8 | [1] |
| WM 02.138 | ATCC 24065; NIH 112 | USA | Clin | <1949 | B/α | 18 | 9 | 5 | 14 | 6 | 28 | 26 | 95 | 0.25 | 2 | 0.25 | 0.125 | 0.125 | 16 | [1] |
| WM 11.943 | B9146 | USA | Clin | 2011 | B/**a** | 18 | 12 | 5 | 14 | 4 | 28 | 26 | 140 | 0.25 | 2 | 0.125 | 0.06 | 0.125 | 8 | [3] |
| WM 02.139 | NIH 198 | USA | Clin | <1971 | B/α | 18 | 3 | 1 | 35 | 6 | 28 | 27 | 93 | 0.25 | 4 | 0.06 | 0.03 | 0.06 | 4 | [1] |
| WM 11.123 | JS94 | USA | Vet | 2011 | B/**a** | 18 | 3 | 1 | 35 | 6 | 40 | 27 | 148 | 0.5 | 2 | 0.125 | 0.06 | 0.125 | 8 | [1] |
| WM 10.186 | JS69 | USA | Vet | 2006 | B/α | 18 | 3 | 1 | 3 | 34 | 40 | 29 | 118 | 0.25 | 0.5 | 0.03 | 0.015 | 0.03 | 2 | [1] |
| WM 11.938 | B8262 | USA | Clin | 1992 | B/α | 18 | 3 | 1 | 3 | 34 | 38 | 19 | 78 | 0.25 | 1 | 0.015 | 0.015 | ≤0.015 | 1 | [2] |
| WM 11.63 | PWQ 1098 | AUS | Clin | 2011 | B/α | 18 | 3 | 1 | 34 | 34 | 38 | 19 | 144 | ≤0.12 | 1 | 0.06 | 0.06 | 0.03 | 8 | WM |
| WM 11.941 | B8965 | USA | Clin | 2010 | B/**a** | 18 | 3 | 1 | 3 | 20 | 28 | 25 | 112 | 0.25 | 1 | 0.06 | 0.03 | 0.03 | 4 | [2] |
| WM 11.948 | B9315 | USA | Clin | 2011 | B/α | 18 | 3 | 1 | 3 | 17 | 40 | 19 | 138 | 0.25 | 1 | 0.03 | 0.015 | ≤0.015 | 4 | [3] |
| WM 11.945 | B9151 | USA | Clin | 2011 | B/α | 18 | 3 | 1 | 3 | 17 | 40 | 19 | 138 | ≤0.12 | 1 | 0.03 | 0.015 | 0.03 | 4 | [3] |
| WM 11.112 | H0058-I-2442 | COL | Clin | 2005 | B/α | 18 | 3 | 1 | 3 | 17 | 28 | 19 | 146 | 0.5 | 4 | 0.125 | 1 | 0.125 | 128 | [5] |
| WM 11.118 | H0058-I-2961 | COL | Clin | 2008 | B/α | 18 | 3 | 1 | 3 | 17 | 28 | 19 | 146 | 0.5 | 2 | 0.125 | 0.06 | 0.125 | 8 | [5] |
| WM 11.131 | JS102 | USA | Vet | 2011 | B/α | 18 | 3 | 1 | 3 | 17 | 28 | 19 | 146 | 0.5 | 4 | 0.06 | 0.015 | 0.03 | 4 | JS |
| WM 1699 | HM136353; LA 290 | PRY | Clin | 1999 | C/α | 20 | 23 | 5 | 23 | 4 | 29 | 21 | 67 | 0.5 | 4 | 0.06 | 0.03 | 0.03 | 4 | [4] |
| WM 2220 | ATCC 32608; NIH 191 | USA | Clin | 1975 | C/**a** | 29 | 12 | 11 | 10 | 4 | 28 | 22 | 68 | ≤0.12 | 1 | 0.06 | 0.03 | 0.03 | 4 | [1] |
| WM 2423 | CN043 | NZL | Clin | ND | C/**a** | 29 | 12 | 11 | 10 | 4 | 28 | 22 | 68 | ≤0.12 | 1 | 0.06 | 0.03 | 0.03 | 4 | [4] |
| WM 09.44 | 06-3303 | USA | Vet | 2006 | C/**a** | 29 | 12 | 11 | 38 | 4 | 28 | 22 | 62 | 0.25 | 1 | 0.06 | 0.03 | 0.03 | 4 | [1] |
| WM 10.121 | 10-02-697 | USA | Vet | 2010 | C/**a** | 29 | 12 | 11 | 2 | 4 | 28 | 22 | 77 | 0.5 | 4 | 0.125 | 0.125 | 0.03 | 32 | [1] |
| WM 10.179 | JS58 | USA | Vet | 2006 | C/α | 29 | 12 | 11 | 2 | 36 | 28 | 21 | 117 | 0.25 | 0.5 | 0.125 | 0.03 | 0.06 | 4 | [1] |
| WM 11.40 | JS91 | USA | Vet | 2011 | C/**a** | 29 | 12 | 11 | 9 | 23 | 28 | 22 | 55 | 0.5 | 1 | 0.06 | 0.06 | 0.03 | 4 | [1] |
| WM 2063 | LA 594; H0058-I-792 | COL | Clin | 1998 | C/α | 29 | 9 | 5 | 9 | 4 | 28 | 21 | 85 | 0.25 | 1 | 0.06 | 0.03 | ≤0.015 | 4 | [5] |
| WM 1666 | UCLA381C | USA | Clin | <1965 | C/α | 29 | 9 | 5 | 2 | 4 | 28 | 19 | 83 | 0.25 | 1 | 0.06 | 0.03 | 0.03 | 8 | [1] |
| WM 1667 | UCLA384C | USA | Clin | <1965 | C/α | 29 | 9 | 5 | 2 | 23 | 28 | 26 | 84 | 0.25 | 2 | 0.125 | 0.125 | 0.125 | 16 | [3] |
| WM 10.17 | 09-11987 | USA | Vet | 2009 | C/α | 29 | 9 | 5 | 14 | 4 | 28 | 26 | 76 | 0.5 | 2 | 0.125 | 0.03 | 0.06 | 16 | [1] |
| WM 183 | ATCC 34880; NIH 312 | USA | Clin | <1971 | C/α | 29 | 9 | 18 | 2 | 23 | 41 | 21 | 86 | 0.25 | 4 | 0.06 | 0.06 | ≤0.015 | 8 | [1] |
| WM 11.937 | B8260 | USA | Vet | 2009 | C/α | 29 | 9 | 18 | 2 | 4 | 28 | 19 | 61 | ≤0.12 | 0.5 | 0.25 | 0.25 | 2 | 64 | [2] |
| WM 11.950 | B9351 | USA | Vet | 2011 | C/α | 29 | 9 | 11 | 2 | 4 | 28 | 21 | 142 | 0.25 | 1 | 0.125 | 0.06 | 0.125 | 8 | [3] |
| WM 1665 | UCLA380C | USA | Clin | <1965 | C/α | 29 | 9 | 11 | 2 | 6 | 28 | 21 | 66 | 0.25 | 1 | 0.125 | 0.06 | 0.03 | 8 | [4] |
| WM 1846 | BV19; LA 382 | VEN | Clin | 1997 | C/α | 29 | 7 | 5 | 2 | 6 | 28 | 21 | 65 | 0.25 | 1 | 0.03 | 0.03 | 0.03 | 2 | [4] |
| WM 2158 | H0058-I-604; LA 644 | COL | Env | 1997 | C/α | 29 | 7 | 5 | 2 | 6 | 28 | 21 | 65 | 0.25 | 4 | 0.06 | 0.03 | 0.03 | 8 | [4] |
| WM 1814 | LA 402; INDRE 5619 | MEX | Clin | 1982 | C/α | 29 | 7 | 5 | 2 | 4 | 28 | 21 | 79 | 0.5 | 2 | 0.25 | 0.125 | 0.125 | 16 | LC |
| WM 1823 | LA 411; INDRE 5628 | MEX | Clin | 1991 | C/α | 29 | 7 | 5 | 2 | 4 | 28 | 21 | 79 | 0.25 | 4 | 0.125 | 0.125 | 0.06 | 16 | LC |
| WM 1916 | H0058-I-953; Col-E6 | COL | Env | 1999 | C/α | 29 | 7 | 5 | 2 | 4 | 28 | 21 | 79 | 2 | 2 | 0.25 | 0.25 | 0.125 | 32 | PE |
| WM 1962 | LA 487; H0058-I-78 | COL | Clin | 1989 | C/α | 29 | 7 | 5 | 2 | 4 | 28 | 21 | 79 | 0.5 | 4 | 0.125 | 0.125 | 0.125 | 16 | PE |
| WM 11.7 | H0058-I-608 | COL | Env | 1997 | C/α | 29 | 7 | 5 | 2 | 4 | 28 | 21 | 79 | ≤0.12 | 0.5 | 0.06 | 0.03 | 0.03 | 4 | PE |
| WM 11.17 | H0058-I-3299 | COL | Env | 2010 | C/α | 29 | 7 | 5 | 2 | 4 | 28 | 21 | 79 | 0.25 | 2 | 0.125 | 0.125 | 0.125 | 16 | PE |
| WM 11.18 | H0058-I-3301 | COL | Env | 2010 | C/α | 29 | 7 | 5 | 2 | 4 | 28 | 21 | 79 | 0.25 | 2 | 0.125 | 0.125 | 0.125 | 16 | PE |
| WM 11.19 | H0058-I-3304 | COL | Env | 2010 | C/α | 29 | 7 | 5 | 2 | 4 | 28 | 21 | 79 | 0.25 | 0.5 | 0.125 | 0.06 | 0.06 | 8 | PE |
| WM 11.20 | H0058-I-3306 | COL | Env | 2010 | C/α | 29 | 7 | 5 | 2 | 4 | 28 | 21 | 79 | 0.25 | 2 | 0.125 | 0.06 | 0.06 | 8 | PE |
| WM 11.105 | H0058-I-2023 | COL | Clin | 2003 | C/α | 29 | 7 | 5 | 2 | 4 | 28 | 21 | 79 | 0.5 | 4 | 0.06 | 0.5 | 0.03 | 128 | [5] |
| WM 11.106 | H0058-I-2086 | COL | Clin | 2004 | C/α | 29 | 7 | 5 | 2 | 4 | 28 | 21 | 79 | 0.5 | 4 | 0.125 | 0.06 | 0.125 | 8 | [5] |
| WM 11.952 | B9370 | USA | Clin | 2011 | C/α | 29 | 7 | 5 | 2 | 4 | 28 | 21 | 79 | 0.25 | 0.5 | 0.125 | 0.06 | 0.125 | 8 | [3] |
| WM 3068 | LA 680; H0058-I-826 | COL | Env | 1998 | C/α | 29 | 7 | 5 | 2 | 4 | 28 | 21 | 79 | ≤0.12 | 1 | 0.125 | 0.06 | 0.06 | 8 | PE |
| WM 3069 | LA 681; H0058-I-859 | COL | Env | 1998 | C/α | 29 | 7 | 5 | 2 | 4 | 28 | 21 | 79 | 0.25 | 2 | 0.125 | 0.06 | 0.125 | 16 | [4] |
| WM 3071 | LA 683; H0058-I-871 | COL | Env | 1999 | C/α | 29 | 7 | 5 | 2 | 4 | 28 | 21 | 79 | 0.25 | 2 | 0.125 | 0.06 | 0.125 | 16 | PE |
| WM 3073 | LA 686; H0058-I-964 | COL | Env | 1999 | C/α | 29 | 7 | 5 | 2 | 4 | 28 | 21 | 79 | 0.25 | 2 | 0.125 | 0.06 | 0.06 | 8 | [4] |
| WM 2148 | LA 634; H0058-I-559 | COL | Env | 1997 | C/α | 29 | 7 | 1 | 2 | 4 | 28 | 21 | 80 | 0.25 | 2 | 0.06 | 0.03 | 0.06 | 8 | PE |
| WM 2169 | LA 655; H0058-I-617 | COL | Env | 1998 | C/α | 29 | 7 | 1 | 2 | 4 | 28 | 21 | 80 | 0.25 | 2 | 0.06 | 0.125 | 0.03 | 4 | PE |
| WM 2179 | LA 665; H0058-I-733 | COL | Env | 1998 | C/α | 29 | 7 | 1 | 2 | 4 | 28 | 21 | 80 | 0.25 | 2 | 0.125 | 0.06 | 0.125 | 8 | PE |
| WM 2170 | LA 656; H0058-I-618 | COL | Env | 1997 | C/α | 29 | 7 | 1 | 2 | 4 | 28 | 21 | 80 | 0.25 | 2 | 0.125 | 0.25 | 0.125 | 8 | PE |
| WM 06.38 | B4546 | USA | Clin | ND | C/**a** | 29 | 7 | 1 | 9 | 4 | 28 | 21 | 81 | 0.25 | 2 | 0.06 | 0.03 | 0.03 | 4 | [4] |
| WM 2173 | LA 659; H0058-I-682 | COL | Env | 1998 | C/α | 29 | 7 | 23 | 2 | 4 | 28 | 21 | 82 | 0.25 | 2 | 0.125 | 0.06 | 0.06 | 8 | PE |
| WM 2177 | LA 663; H0058-I-731 | COL | Env | 1998 | C/α | 29 | 7 | 23 | 2 | 4 | 28 | 21 | 82 | 0.25 | 2 | 0.125 | 0.06 | 0.06 | 8 | [4] |
| WM 2182 | LA 668; H0058-I-760 | COL | Env | 1998 | C/α | 29 | 7 | 23 | 2 | 4 | 28 | 21 | 82 | 0.25 | 2 | 0.125 | 0.06 | 0.125 | 8 | PE |
| WM 2188 | LA 674; H0058-I-818 | COL | Env | 1998 | C/α | 29 | 7 | 23 | 2 | 4 | 28 | 21 | 82 | 0.25 | 2 | 0.125 | 0.125 | 0.125 | 16 | PE |
| WM 11.946 | B9237, 2011793521 | USA | Vet | 2011 | C/α | 29 | 7 | 5 | 39 | 4 | 28 | 19 | 141 | 0.25 | 1 | 0.06 | 0.125 | 0.06 | 8 | [3] |
| WM 1811 | LA 399; INDRE 5618 | MEX | Clin | 1980 | C/α | 35 | 3 | 5 | 22 | 20 | 28 | 28 | 99 | ≤0.12 | 1 | 0.03 | 0.03 | ≤0.015 | 2 | CC |
| WM 1812 | LA 400; INDRE 5617 | MEX | Clin | 1980 | C/α | 35 | 3 | 5 | 22 | 20 | 28 | 28 | 99 | 0.25 | 2 | 0.06 | 0.06 | 0.03 | 4 | CC |
| WM 11.32^f^ | JS81 | USA | Vet | 2010 | B/α | 43 | 31 | 64 | 43 | 32 | 64 | 18 | 114 | 0.25 | 1 | 0.06 | 0.03 | ≤0.015 | 2 | [1] |
| WM 2004^f^ | H0058-I-256 | COL | Clin | 1994 | B/α | 43 | 31 | 63 | 43 | 32 | 39 | 18 | 64 | 0.5 | 4 | 0.125 | 0.06 | 0.125 | 4 | PE |
| WM 2041^f^ | H0058-I-642; LA 568 | COL | Clin | 1997 | B/α | 43 | 31 | 63 | 43 | 32 | 39 | 18 | 64 | 0.25 | 4 | 0.06 | 0.03 | 0.03 | 4 | PE |
| WM 2042^f^ | H0058-I-645 | COL | Clin | 1997 | B/α | 43 | 31 | 63 | 43 | 32 | 39 | 18 | 64 | 0.5 | 8 | 0.06 | 0.06 | 0.125 | 2 | PE |
| WM 1804^f^ | INDRE5606; LA 392 | MEX | Clin | <2000 | C/**a** | 43 | 28 | 61 | 41 | 31 | 39 | 17 | 101 | 0.25 | 1 | 0.125 | 0.03 | 0.125 | 4 | CC |
| WM 1802^f^ | INDRE5604; LA 390 | MEX | Clin | <2000 | C/**a** | 42 | 28 | 61 | 41 | 31 | 39 | 17 | 100 | 0.25 | 1 | 0.03 | ≤0.008 | ≤0.015 | 1 | CC |
| WM 179 | CBS 10078 | AUS | Clin | 1993 | B/α | 16 | 5 | 3 | 5 | 5 | 32 | 12 | 51 | ≤0.12 | 0.5 | 0.12 | 0.03 | 0.12 | 4 | [6] |
| WM 178 | CBS 10082 | AUS | Clin | 1991 | B/α | 1 | 17 | 16 | 16 | 14 | 19 | 7 | 21 | 0.5 | 2 | 0.06 | 0.03 | 0.03 | 4 | [6] |
| WM 779 | CBS 10101 | ZAF | Vet | 1995 | C/α | 17 | 10 | 8 | 18 | 3 | 37 | 11 | 70 | 0.5 | 1 | 0.06 | 0.06 | 0.06 | 0.5 | [6] |

^a^Isolates from AUS: Australia, COL: Colombia, GTM: Guatemala, MEX: Mexico, NZL: New Zealand, PRY: Paraguay, USA: United States, VEN: Venezuela and ZAF: South Africa.

^b^Clin: Clinical; Vet: Veterinary; Env: Environmental

^c^Serotype of the isolates identified simultaneously by agglutination using the Crypto-Check (Iatron Laboratories, Tokyo, Japan) and *CAP59*=RFLP [61] are underlined (n=53).

^d^Minimum inhibitory concentrations (MIC) to AMB: Amphotericin-B; FC: 5-Fluorocytosine; PCZ: Posaconazole, VCZ: Voriconazole; ITZ: Itraconazole; FCZ: Fluconazole

^e^Strains deposited by: CC: Cudberto Contreras-Peres; JS: Jane Sykes; LC: Laura Rocío Castañón-Olivares; PE: Patricia Escandón; SL: Shawn Lockhart; WM: Wieland Meyer.

^f^Identified as VGIV by *URA5*-RFLP

**S1 References**

1. Singer LM, Meyer W, Firacative C, Thompson GR 3rd, Samitz E, Sykes JE. Antifungal drug susceptibility and phylogenetic diversity among *Cryptococcus* isolates from dogs and cats in North America. J Clin Microbiol. 2014;52: 2061-2070.
2. Walraven CJ, Gerstein W, Hardison SE, Wormley F, Lockhart SR, Harris JR, et al. Fatal disseminated *Cryptococcus gattii* infection in New Mexico. PLoS One. 2011;6: e28625.
3. Lockhart SR, Iqbal N, Harris JR, Grossman NT, DeBess E, Wohrle R, et al. *Cryptococcus gattii* in the United States: genotypic diversity of human and veterinary isolates. PLoS One. 2013;8: e74737.
4. Firacative C, Trilles L, Meyer W. MALDI-TOF MS enables the rapid identification of the major molecular types within the *Cryptococcus neoformans/C. gattii* species complex. PLoS One. 2012;7: e37566.
5. Lizarazo J, Escandón P, Agudelo CI, Firacative C, Meyer W, Castañeda E. Retrospective study of the epidemiology and clinical manifestations of *Cryptococcus gattii* infections in Colombia from 1997-2011. PLoS Negl Trop Dis. 2014;8: e3272.
6. Ngamskulrungroj P, Gilgado F, Faganello J, Litvintseva AP, Leal AL, Tsui KM, et al. Genetic diversity of the *Cryptococcus* species complex suggests that *Cryptococcus gattii* deserves to have varieties. PLoS One. 2009;4: e5862.
