# Supplementary material for: MLST and Whole-Genome-Based Population Analysis of Cryptococcus gattii VGIII Links Clinical, Veterinary and Environmental Strains, and Reveals Divergent Serotype Specific Sub-populations and Distant Ancestors
Source: PLoS Negl Trop Dis. 2016 Aug 5;10(8):e0004861. doi: 10.1371/journal.pntd.0004861 (PMC4975453; doi:10.1371/journal.pntd.0004861)
Supplement: S2 Table — GenBank accession numbers for the allele types of Cryptococcus gattii molecular type VGIII isolates. (DOC) [file pntd.0004861.s002.doc]

**S2 Table. Deposited allele sequences.** GenBank accession numbers for the allele types of the studied *Cryptococcus gattii* molecular type VGIII isolates.

| **Locus** | **Allele** | **GenBank Accession No** |
| --- | --- | --- |
| ***CAP59*** | *CAP59* allele # 18 | JX840782 |
|  | *CAP59* allele # 20 | JX840783 |
|  | *CAP59* allele # 29 | JX840784 |
|  | *CAP59* allele # 35 | JX840785 |
|  | *CAP59* allele # 42 | JX840786 |
|  | *CAP59* allele # 43 | JX840787 |
| ***GPD1*** | *GPD1* allele # 3 | JX840788 |
|  | *GPD1* allele # 7 | JX840789 |
|  | *GPD1* allele # 9 | JX840790 |
|  | *GPD1* allele # 12 | JX840791 |
|  | *GPD1* allele # 18 | JX840792 |
|  | *GPD1* allele # 23 | JX840793 |
|  | *GPD1* allele # 28 | JX840794 |
|  | *GPD1* allele # 31 | JX840795 |
| **IGS1** | IGS1 allele # 1 | JX840796 |
|  | IGS1 allele # 5 | JX840797 |
|  | IGS1 allele # 11 | JX840798 |
|  | IGS1 allele # 14 | JX840799 |
|  | IGS1 allele # 18 | JX840800 |
|  | IGS1 allele # 23 | JX840801 |
|  | IGS1 allele # 61 | JX840802 |
|  | IGS1 allele # 63 | JX840803 |
|  | IGS1 allele # 64 | JX840804 |
| ***LAC1*** | *LAC1* allele # 2 | JX840805 |
|  | *LAC1* allele # 3 | JX840806 |
|  | *LAC1* allele # 9 | JX840807 |
|  | *LAC1* allele # 10 | JX840808 |
|  | *LAC1* allele # 14 | JX840809 |
|  | *LAC1* allele # 15 | JX840810 |
|  | *LAC1* allele # 20 | JX840811 |
|  | *LAC1* allele # 22 | JX840812 |
|  | *LAC1* allele # 23 | JX840813 |
|  | *LAC1* allele # 32 | JX840814 |
|  | *LAC1* allele # 33 | JX840815 |
|  | *LAC1* allele # 34 | JX840816 |
|  | *LAC1* allele # 35 | JX840817 |
|  | *LAC1* allele # 38 | JX840818 |
|  | *LAC1* allele # 39 | JX840819 |
|  | *LAC1* allele # 41 | JX840820 |
|  | *LAC1* allele # 43 | JX840821 |
| ***PLB1*** | *PLB1* allele # 4 | JX840822 |
|  | *PLB1* allele # 6 | JX840823 |
|  | *PLB1* allele # 17 | JX840824 |
|  | *PLB1* allele # 20 | JX840825 |
|  | *PLB1* allele # 21 | JX840826 |
|  | *PLB1* allele # 23 | JX840827 |
|  | *PLB1* allele # 31 | JX840828 |
|  | *PLB1* allele # 32 | JX840829 |
|  | *PLB1* allele # 34 | JX840830 |
|  | *PLB1* allele # 35 | JX840831 |
|  | *PLB1* allele # 36 | JX840832 |
| ***SOD1*** | *SOD1* allele # 28 | JX840833 |
|  | *SOD1* allele # 29 | JX840834 |
|  | *SOD1* allele # 38 | JX840835 |
|  | *SOD1* allele # 39 | JX840836 |
|  | *SOD1* allele # 40 | JX840837 |
|  | *SOD1* allele # 41 | JX840838 |
|  | *SOD1* allele # 48 | JX840839 |
|  | *SOD1* allele # 64 | JX840840 |
| ***URA5*** | *URA5* allele # 17 | JX840841 |
|  | *URA5* allele # 18 | JX840842 |
|  | *URA5* allele # 19 | JX840843 |
|  | *URA5* allele # 21 | JX840844 |
|  | *URA5* allele # 22 | JX840845 |
|  | *URA5* allele # 23 | JX840846 |
|  | *URA5* allele # 25 | JX840847 |
|  | *URA5* allele # 26 | JX840848 |
|  | *URA5* allele # 27 | JX840849 |
|  | *URA5* allele # 28 | JX840850 |
|  | *URA5* allele # 29 | JX840851 |
