# Supplementary material for: MLST and Whole-Genome-Based Population Analysis of Cryptococcus gattii VGIII Links Clinical, Veterinary and Environmental Strains, and Reveals Divergent Serotype Specific Sub-populations and Distant Ancestors
Source: PLoS Negl Trop Dis. 2016 Aug 5;10(8):e0004861. doi: 10.1371/journal.pntd.0004861 (PMC4975453; doi:10.1371/journal.pntd.0004861)
Supplement: S3 Table — Diversity of the sequence types (STs) of Cryptococcus gattii molecular type VGIII isolates (n = 122). (DOC) [file pntd.0004861.s003.doc]

**S3 Table**. **Genotypic diversity.** Diversity of the sequence types (STs) of the studied *Cryptococcus gattii* molecular type VGIII isolates (n=122).

| **Population group** | **n of isolates** | **n of STs** | ***D***^a^ |
| --- | --- | --- | --- |
| Total | 122 | 55 | 0.061 |
| *Country* | | | |
| Mexico | 14 | 11 | 0.102 |
| USA | 66 | 37 | 0.109 |
| Colombia | 37 | 9 | 0.196 |
| Other countries (n=5) | 5 | 5 | ND |
| *Source* | | | |
| Clinical | 56 | 35 | 0.048 |
| Environmental | 28 | 6 | 0.055 |
| Veterinary | 38 | 19 | 0.084 |
| *Serotype* | | | |
| Serotype C | 47 | 23 | 0.056 |
| Serotype B | 75 | 32 | 0.105 |
| *Mating type* | | | |
| Mating type a | 23 | 13 | 0.008 |
| Mating type alpha | 99 | 42 | 0.085 |

^a^*D*: Simpsons diversity index

ND: No determined
