# Supplementary material for: MLST and Whole-Genome-Based Population Analysis of Cryptococcus gattii VGIII Links Clinical, Veterinary and Environmental Strains, and Reveals Divergent Serotype Specific Sub-populations and Distant Ancestors
Source: PLoS Negl Trop Dis. 2016 Aug 5;10(8):e0004861. doi: 10.1371/journal.pntd.0004861 (PMC4975453; doi:10.1371/journal.pntd.0004861)
Supplement: S4 Table — Comparison of MIC values for significant differences between Cryptococcus gattii molecular type VGIII isolates (n = 122). (DOC) [file pntd.0004861.s004.doc]

**S4 Table.** **Antifungal susceptibility differences.** Comparison of MIC values for significant differences between the studied *Cryptococcus gattii* molecular type VGIII isolates (n=122).

|  | **Antifungal drug^a^** | | | | | |
| --- | --- | --- | --- | --- | --- | --- |
| **Groups compared (n)** | **AMB** | **FC** | **PCZ** | **VCZ** | **ITZ** | **FCZ** |
| Serotype B (75) vs. C (47) | *p* = .1153 | *p* = .0397^*^ | *p* < .0001^****^ | *p* < .0001^****^ | *p* = .0031^**^ | *p* < .0001^****^ |
| Mating type **a** (23) vs. alpha (99) | *p* = .4388 | *p* = .7295 | *p* = .7743 | *p* = .2861 | *p* = .9548 | *p* = .2594 |
| Clinical (56) vs. Environmental (28) | *p* = .2955 | *p* = .3717 | *p* = .0154^*^ | *p* = .0596 | *p* = .0250^*^ | *p* = .0265^*^ |
| Clinical (56) vs. Veterinary (38) | *p* = .0326^*^ | *p* = .2001 | *p* = .0436^*^ | *p* = .0323^*^ | *p* = .0494^*^ | *p* = .0410^*^ |
| Environmental (28) vs. Veterinary (38) | *p* = .0004^***^ | *p* = .0320^*^ | *p* = .0001^***^ | *p* = .0006^***^ | *p* < .0001^****^ | *p* < .0001^****^ |

^a^AMB: Amphotericin-B; FC: 5-Fluorocytosine; PCZ: Posaconazole, VCZ: Voriconazole; ITZ: Itraconazole; FCZ: Fluconazole
