# Supplementary material for: MLST and Whole-Genome-Based Population Analysis of Cryptococcus gattii VGIII Links Clinical, Veterinary and Environmental Strains, and Reveals Divergent Serotype Specific Sub-populations and Distant Ancestors
Source: PLoS Negl Trop Dis. 2016 Aug 5;10(8):e0004861. doi: 10.1371/journal.pntd.0004861 (PMC4975453; doi:10.1371/journal.pntd.0004861)
Supplement: S5 Table — Distribution of MIC values of Cryptococcus gattii molecular type VGIII isolates by serotype. (DOC) [file pntd.0004861.s005.doc]

**S5 Table.** **MIC values per serotype.** Distribution of MIC values of the studied *Cryptococcus gattii* molecular type VGIII isolates by serotype.

|  |  | **No. of isolates at MIC value (μg/ml)^a^** | | | | | | | | | | | | | | |
| --- | --- | --- | --- | --- | --- | --- | --- | --- | --- | --- | --- | --- | --- | --- | --- | --- |
| **Antifungal^b^** | **Serotype** | **≤0.008** | **0.015** | **0.03** | **0.06** | **0.12** | **0.25** | **0.5** | **1** | **2** | **4** | **8** | **16** | **32** | **64** | **128** |
| AMB | Serotype B (75) |  |  |  |  | 7 | 43 | 24 | 1 |  |  |  |  |  |  |  |
|  | Serotype C (47) |  |  |  |  | 6 | 32 | 8 |  | 1 |  |  |  |  |  |  |
|  | All |  |  |  |  | 13 | 75 | 32 | 1 | 1 |  |  |  |  |  |  |
| FC | Serotype B (75) |  |  |  |  |  |  | 3 | 21 | 24 | 22 | 5 |  |  |  |  |
|  | Serotype C (47) |  |  |  |  |  |  | 5 | 14 | 20 | 8 |  |  |  |  |  |
|  | All |  |  |  |  |  |  | 8 | 35 | 44 | 30 | 5 |  |  |  |  |
| PCZ | Serotype B (75) |  | 5 | 18 | 37 | 12 | 3 |  |  |  |  |  |  |  |  |  |
|  | Serotype C (47) |  |  | 3 | 16 | 25 | 3 |  |  |  |  |  |  |  |  |  |
|  | All |  | 5 | 21 | 53 | 37 | 6 |  |  |  |  |  |  |  |  |  |
| VCZ | Serotype B (75) | 1 | 19 | 35 | 16 | 3 |  |  | 1 |  |  |  |  |  |  |  |
|  | Serotype C (47) | 1 | 0 | 15 | 17 | 10 | 3 | 1 |  |  |  |  |  |  |  |  |
|  | All | 2 | 19 | 50 | 33 | 13 | 3 | 1 | 1 |  |  |  |  |  |  |  |
| ITZ | Serotype B (75) |  | 21 | 28 | 10 | 15 | 1 |  |  |  |  |  |  |  |  |  |
|  | Serotype C (47) |  | 4 | 15 | 11 | 16 |  |  |  | 1 |  |  |  |  |  |  |
|  | All |  | 25 | 43 | 21 | 31 | 1 |  |  | 1 |  |  |  |  |  |  |
| FCZ | Serotype B (75) |  |  |  |  |  |  |  | 3 | 21 | 33 | 14 | 3 |  |  | 1 |
|  | Serotype C (47) |  |  |  |  |  |  |  | 1 | 2 | 12 | 18 | 10 | 2 | 1 | 1 |
|  | All |  |  |  |  |  |  |  | 4 | 23 | 45 | 32 | 13 | 2 | 1 | 2 |

^a^The modal MIC for each distribution is underlined

^b^AMB: Amphotericin-B; FC: 5-Fluorocytosine; PCZ: Posaconazole, VCZ: Voriconazole; ITZ: Itraconazole; FCZ: Fluconazole
