# Supplementary figures and images for: MLST and Whole-Genome-Based Population Analysis of Cryptococcus gattii VGIII Links Clinical, Veterinary and Environmental Strains, and Reveals Divergent Serotype Specific Sub-populations and Distant Ancestors
Source: PLoS Negl Trop Dis. 2016 Aug 5;10(8):e0004861. doi: 10.1371/journal.pntd.0004861 (PMC4975453; doi:10.1371/journal.pntd.0004861)

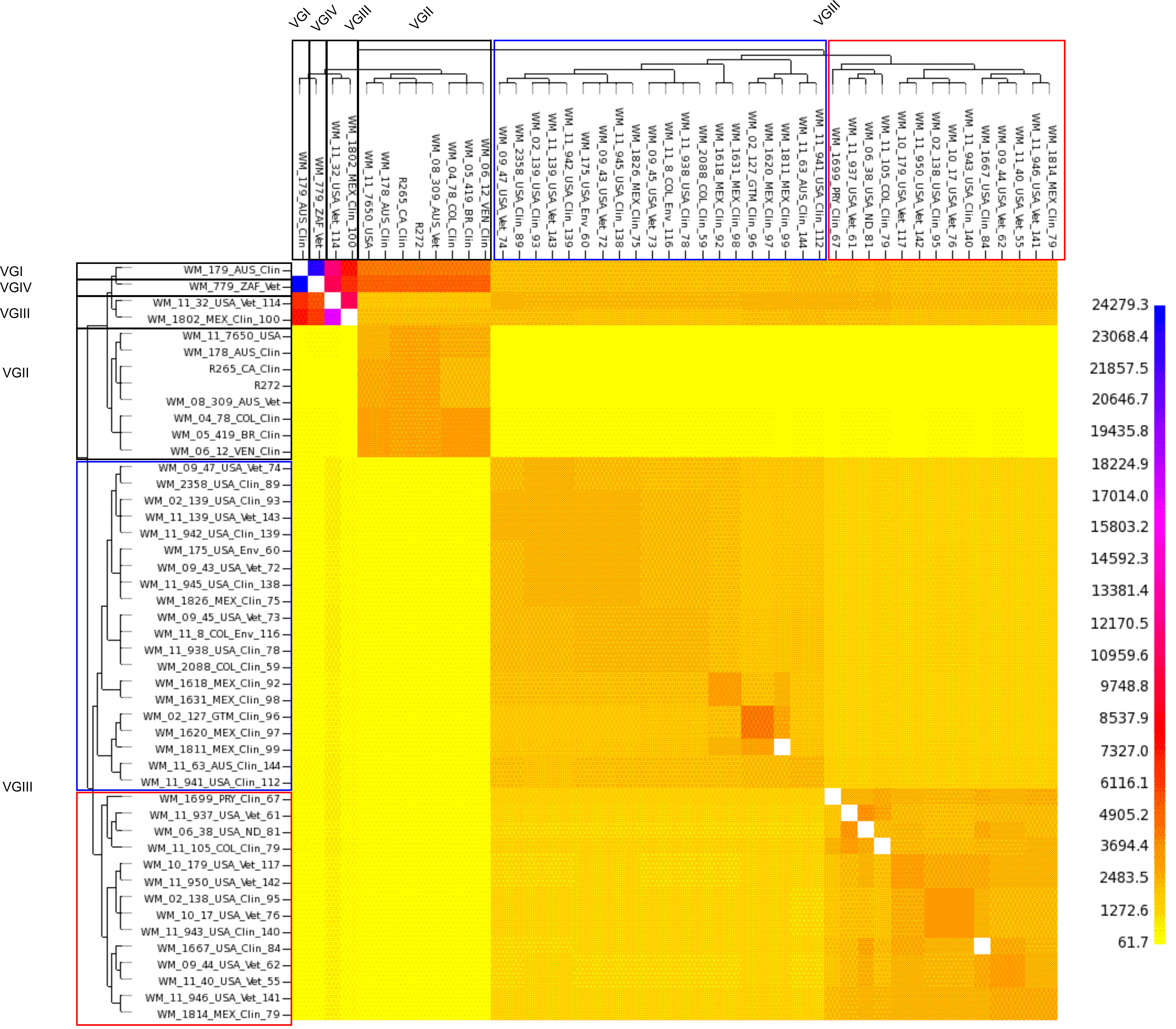

Supplement: S2 Fig — To identify the population structure and genome exchange events between the VGIII groups and the remaining C. gattii molecular types, fineStructure analysis was performed using a SNP matrix (i). Whole-genome SNP data were reduced to a pairwise similarity matrix. The x-axis represents the strain as a “donor” and the y-axis represents the strain as a “recipient” of genomic regions. The scale bar represents the number of shared genome regions with blue being the greatest amount of sharing and yellow being the least. Blue and red boxes represent the main VGIII serotype B and serotype C isolates, respectively. (TIF) [file pntd.0004861.s007.tif]

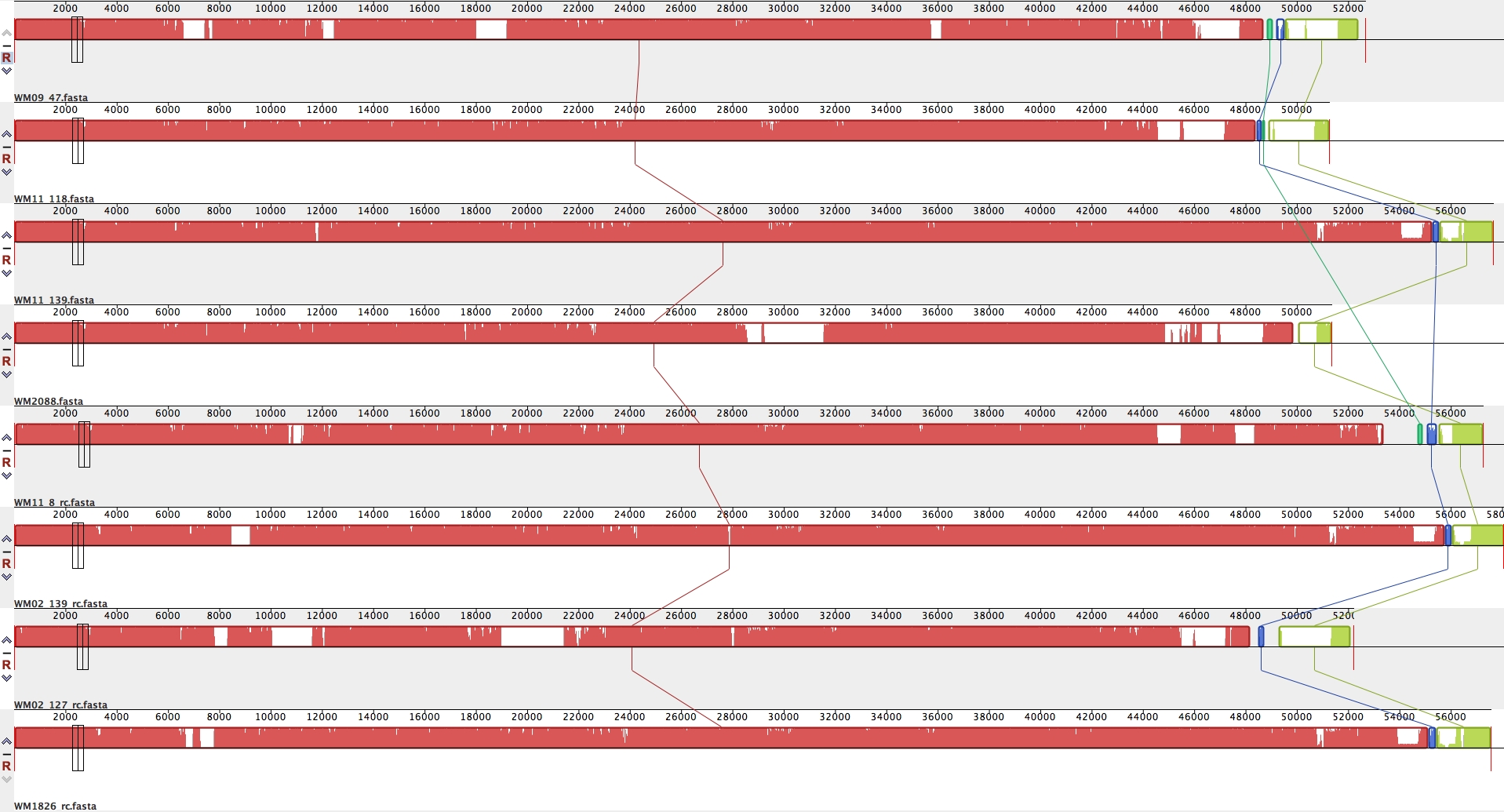

Supplement: S4 Fig — High virulent: WM 09.47, WM 11.118, WM 11.139, WM 2088, and low virulent: WM 11.8, WM 02.1`39, WM 02.127, WM 18260 C. gattii VGIII strains. (JPEG) [file pntd.0004861.s009.jpeg]
